# Ursodeoxycholic acid in intrahepatic cholestasis of pregnancy: a systematic review and individual participant data meta-analysis

**DOI:** 10.1016/S2468-1253(21)00074-1

**Published:** 2021-04-27

**Authors:** Caroline Ovadia, Jenna Sajous, Paul T Seed, Kajol Patel, Nicholas J Williamson, George Attilakos, Francesco Azzaroli, Yannick Bacq, Linoy Batsry, Kelsey Broom, Romana Brun-Furrer, Laura Bull, Jenny Chambers, Yue Cui, Min Ding, Peter H Dixon, Maria C Estiú, Fergus W Gardiner, Victoria Geenes, Monika Grymowicz, Berrin Günaydin, William M Hague, Christian Haslinger, Yayi Hu, Ugo Indraccolo, Alexander Juusela, Stefan C Kane, Ayse Kebapcilar, Levent Kebapcilar, Katherine Kohari, Jūratė Kondrackienė, Maria P H Koster, Richard H Lee, Xiaohua Liu, Anna Locatelli, Rocio I R Macias, Riza Madazli, Agata Majewska, Kasia Maksym, Jessica A Marathe, Adam Morton, Martijn A Oudijk, Deniz Öztekin, Michael J Peek, Andrew H Shennan, Rachel M Tribe, Valeria Tripodi, Naciye Türk Özterlemez, Tharni Vasavan, L F Audris Wong, Yoav Yinon, Qianwen Zhang, Keren Zloto, Hanns-Ulrich Marschall, Jim Thornton, Lucy C Chappell, Catherine Williamson

**Affiliations:** a Department of Women and Children's Health, King's College London, London, UK; b Department of Obstetrics and Gynaecology, University College London Hospitals NHS Foundation Trust, London, UK; c Department of Medical and Surgical Sciences, University of Bologna, Bologna, Italy; d Department of Hepatology and Gastroenterology, University Hospital of Tours, Tours, France; e Department of Obstetrics and Gynecology, Sheba Medical Center, Sackler School of Medicine, Tel-Aviv University, Tel-Aviv, Israel; f Bendigo Healthcare Group, Bendigo, VIC, Australia; g Department of Obstetrics, University Hospital Zurich, Zurich, Switzerland; h Department of Medicine and Institute for Human Genetics, University of California, San Francisco, CA, USA; i Women's Health Research Centre, Imperial College London, London, UK; j School of Laboratory Medicine, Chongqing Medical University, Chongqing, China; k Ramón Sardá Mother's and Children's Hospital, Buenos Aires, Argentina; l Royal Flying Doctor Service, Barton, ACT, Australia; m Department of Gynecological Endocrinology, Medical University of Warsaw, Warsaw, Poland; n First Department of Obstetrics and Gynecology, Medical University of Warsaw, Warsaw, Poland; o Department of Anesthesiology and Reanimation, Gazi University School of Medicine, Ankara, Turkey; p Robinson Research Institute, University of Adelaide, Adelaide, SA, Australia; q Department of Obstetrics and Gynecology, West China Second University Hospital, Sichuan University, Chengdu, China; r Maternal-Infantile Department, Complex Operative Unit of Obstetrics and Gynecology Alto Tevere Hospital of Città di Castello, Città di Castello, Italy; s Newark Beth Israel Medical Center, Newark, NJ, USA; t Department of Maternal-Fetal Medicine, Royal Women's Hospital, Parkville, VIC, Australia; u Department of Obstetrics and Gynaecology, University of Melbourne, Melbourne, VIC, Australia; v Department of Gynecology and Obstetrics, Selcuk University, Konya, Turkey; w Department of Internal Medicine, Selcuk University, Konya, Turkey; x Department of Obstetrics, Gynecology and Reproductive Sciences, Yale School of Medicine, New Haven, CT, USA; y Department of Gastroenterology, Lithuanian University of Health Sciences, Kaunas, Lithuania; z Department of Obstetrics and Gynaecology, Erasmus MC, University Medical Center Rotterdam, Netherlands; aa Department of Obstetrics and Gynecology, Keck School of Medicine University of Southern California, Los Angeles, CA, USA; ab Shanghai First Maternity and Infant Hospital, Tongji University School of Medicine, Shanghai, China; ac Department of Obstetrics and Gynecology, University of Milano-Bicocca, Monza, Italy; ad Department of Physiology and Pharmacology, Centro de Investigación Biomédica en Red de Enfermedades Hepáticas y Digestivas, Institute of Biomedical Research of Salamanca, University of Salamanca, Salamanca, Spain; ae Department of Obstetrics and Gynecology, Istanbul University, Cerrahpaşa, Istanbul, Turkey; af Department of Cardiology, Royal Adelaide Hospital, Adelaide, SA, Australia; ag Department of Obstetric Medicine, Mater Health Services Public Hospital, Brisbane, QLD, Australia; ah Department of Obstetrics, Amsterdam University Medical Center, University of Amsterdam, Amsterdam, Netherlands; ai Department of Obstetrics and Gynecology, İzmir Bakircay University, İzmir, Turkey; aj ANU Medical School, College of Health and Medicine, The Australian National University, Canberra, ACT, Australia; ak Facultad de Farmacia y Bioquímica, Universidad de Buenos Aires, Buenos Aires, Argentina; al Department of Women's and Newborn, Gold Coast University Hospital, Southport, QLD, Australia; am Department of Molecular and Clinical Medicine, University of Gothenburg, Gothenburg, Sweden; an Division of Child Health, Obstetrics and Gynaecology, University of Nottingham, Nottingham, UK

## Abstract

**Background:**

Ursodeoxycholic acid is commonly used to treat intrahepatic cholestasis of pregnancy, yet its largest trial detected minimal benefit for a composite outcome (stillbirth, preterm birth, and neonatal unit admission). We aimed to examine whether ursodeoxycholic acid affects specific adverse perinatal outcomes.

**Methods:**

In this systematic review and individual participant data meta-analysis, we searched PubMed, Web of Science, Embase, MEDLINE, CINAHL, Global Health, MIDIRS, and Cochrane without language restrictions for relevant articles published between database inception, and Jan 1, 2020, using search terms referencing intrahepatic cholestasis of pregnancy, ursodeoxycholic acid, and perinatal outcomes. Eligible studies had 30 or more study participants and reported on at least one individual with intrahepatic cholestasis of pregnancy and bile acid concentrations of 40 μmol/L or more. We also included two unpublished cohort studies. Individual participant data were collected from the authors of selected studies. The primary outcome was the prevalence of stillbirth, for which we anticipated there would be insufficient data to achieve statistical power. Therefore, we included a composite of stillbirth and preterm birth as a main secondary outcome. A mixed-effects meta-analysis was done using multi-level modelling and adjusting for bile acid concentration, parity, and multifetal pregnancy. Individual participant data analyses were done for all studies and in different subgroups, which were produced by limiting analyses to randomised controlled trials only, singleton pregnancies only, or two-arm studies only. This study is registered with PROSPERO, CRD42019131495.

**Findings:**

The authors of the 85 studies fulfilling our inclusion criteria were contacted. Individual participant data from 6974 women in 34 studies were included in the meta-analysis, of whom 4726 (67·8%) took ursodeoxycholic acid. Stillbirth occurred in 35 (0·7%) of 5097 fetuses among women with intrahepatic cholestasis of pregnancy treated with ursodeoxycholic acid and in 12 (0·6%) of 2038 fetuses among women with intrahepatic cholestasis of pregnancy not treated with ursodeoxycholic acid (adjusted odds ratio [aOR] 1·04, 95% CI 0·35–3·07; p=0·95). Ursodeoxycholic acid treatment also had no effect on the prevalence of stillbirth when considering only randomised controlled trials (aOR 0·29, 95% CI 0·04–2·42; p=0·25). Ursodeoxycholic acid treatment had no effect on the prevalence of the composite outcome in all studies (aOR 1·28, 95% CI 0·86–1·91; p=0·22), but was associated with a reduced composite outcome when considering only randomised controlled trials (0·60, 0·39–0·91; p=0·016).

**Interpretation:**

Ursodeoxycholic acid treatment had no significant effect on the prevalence of stillbirth in women with intrahepatic cholestasis of pregnancy, but our analysis was probably limited by the low overall event rate. However, when considering only randomised controlled trials, ursodeoxycholic acid was associated with a reduction in stillbirth in combination with preterm birth, providing evidence for the clinical benefit of antenatal ursodeoxycholic acid treatment.

**Funding:**

Tommy's, the Wellcome Trust, ICP Support, and the National Institute for Health Research.

## Introduction

Intrahepatic cholestasis of pregnancy affects 0·3–5·6% of pregnant women, with marked differences by ethnicity.^1^ Affected women develop pruritus and liver dysfunction, with raised serum concentrations of total bile acids and, often, liver aminotransferases.^2^ Increased bile acid peak concentrations (particularly ≥40 μmol/L) are associated with higher rates of adverse perinatal outcomes, including spontaneous preterm birth, meconium-stained amniotic fluid, and neonatal unit admission;^3^, ^4^, ^5^ when bile acid concentrations are 100 μmol/L or more, women have an increased risk of stillbirth (3·44% *vs* 0·28%).^5^, ^6^

**Research in context**

**Evidence before this study**

Pregnancies complicated by intrahepatic cholestasis of pregnancy are known to have an increased risk of perinatal complications, including preterm birth (both spontaneous and clinician-initiated), meconium-stained amniotic fluid, neonatal unit admission, and, for women with peak bile acid concentrations more than 100 μmol/L, stillbirth. Ursodeoxycholic acid is the most used treatment for intrahepatic cholestasis of pregnancy, yet there is no consensus as to its benefit for women or their babies. We searched PubMed, Web of Science, Embase, MEDLINE, CINAHL, Global Health, MIDIRS, and Cochrane for meta-analyses of ursodeoxycholic acid use in intrahepatic cholestasis of pregnancy published between database inception and Aug 1, 2020, using the search terms “meta$analysis”, “cholestasis”, “pregnancy”, and “ursodeoxycholic acid”. There were no language restrictions. Although multiple meta-analyses have been published, most were published before publication of the 2019 PITCHES randomised controlled trial (RCT) by Lucy C Chappell and colleagues. This RCT was of an equivalent size to the sum of all previous trials of ursodeoxycholic acid in intrahepatic cholestasis of pregnancy, many of which only administered ursodeoxycholic acid for limited durations (2–3 weeks), often with treatment unblinded to participants and clinicians. The 2020 Cochrane systematic review of pharmacological treatments for intrahepatic cholestasis of pregnancy included the PITCHES trial, and concluded that ursodeoxycholic acid was able to reduce itching to a minimal degree, serum liver aminotransferase concentrations, and the incidence of meconium-stained amniotic fluid, but the studies of its use for perinatal benefit were not of sufficient quality to provide clear evidence for its use in intrahepatic cholestasis of pregnancy. To our knowledge, no study has reported the effect of ursodeoxycholic acid using individual participant data, or had sufficient statistical power to show any effect of ursodeoxycholic acid on stillbirth.

**Added value of this study**

We did a systematic review and individual participant data meta-analysis, including additional unpublished cohorts of women with intrahepatic cholestasis of pregnancy, to explore how ursodeoxycholic acid treatment impacts adverse perinatal outcomes in intrahepatic cholestasis of pregnancy. Although the study was underpowered to show a statistically significant reduction in the overall prevalence of stillbirth with ursodeoxycholic acid use, we did show that the prevalence of a composite of stillbirth and preterm birth was lower for women treated with ursodeoxycholic acid than for women not treated with ursodeoxycholic acid, when the analysis was restricted to RCTs, with the number needed to treat equalling 15. We also showed that the risk of preterm birth was reduced for women in RCTs with the use of ursodeoxycholic acid, and when considering only singleton pregnancies in all studies.

**Implications of all the available evidence**

Our study shows that women with intrahepatic cholestasis of pregnancy who were treated with ursodeoxycholic acid had lower rates of preterm birth, and a composite outcome of stillbirth and preterm birth, than did women who were not treated with ursodeoxycholic acid. We showed that the benefit of ursodeoxycholic acid treatment on reducing spontaneous preterm birth was statistically significant for women with higher bile acid concentrations (≥40 μmol/L). Adverse outcomes in intrahepatic cholestasis of pregnancy are associated with higher bile acid concentrations, so women with more severe disease are likely to glean the greatest benefit from ursodeoxycholic acid. This study suggests that ursodeoxycholic acid should be offered as part of antenatal treatment for intrahepatic cholestasis of pregnancy, and, particularly, to women with a disease onset before 37 gestational weeks and serum bile acid concentrations of 40 μmol/L or more.

Ursodeoxycholic acid is commonly used for the treatment of intrahepatic cholestasis of pregnancy.^1^ Ursodeoxycholic acid improves biliary flow,^7^ enhances the protective bicarbonate environment on the surface of cholangiocytes,^8^ and protects the liver from bile acid-induced apoptosis.^9^ This therapy has anti-inflammatory actions,^10^ and can reduce the elevation of serum bile acid concentration in the fetus, probably by upregulating placental bile acid export.^11^ As ursodeoxycholic acid is a bile acid, its use results in alteration of the bile acid pool so that it constitutes 60–70% of total bile acids in treated women and replaces more harmful (hydrophobic) bile acids.^12^, ^13^ Although not licensed for use in pregnancy, ursodeoxycholic acid is thought to be safe, with gastrointestinal side-effects being the most common side-effects; however, no difference in the overall rate of side-effects, including serious adverse events, between ursodeoxycholic acid and placebo tablets has been reported.^14^

Studies have shown that ursodeoxycholic acid treatment is associated with reduced pruritus,^15^ but not to a predetermined clinically beneficial degree.^16^ Whether ursodeoxycholic acid improves perinatal outcomes is unclear; contradictory findings, which were dependent on the method of comparison, have been reported from previous meta-analyses of aggregate data of trials of its use.^15^, ^17^, ^18^, ^19^ However, these meta-analyses were limited by study sizes, including 600–700 women from all contributing studies. A randomised controlled trial (RCT) of ursodeoxycholic acid in 605 women with intrahepatic cholestasis of pregnancy showed no improvement in the primary outcome (a composite of perinatal death, preterm birth, and neonatal unit admission) with ursodeoxycholic acid; the incidence of meconium-stained amniotic fluid was the only secondary perinatal outcome to improve (ie, decrease) with ursodeoxycholic acid.^20^ Similarly, a Cochrane review of treatment for intrahepatic cholestasis of pregnancy showed that the evidence for the impact of ursodeoxycholic acid on fetal distress and stillbirth (the principal perinatal outcomes) was uncertain because of limitations in study design and imprecision.^14^ These findings contrast with a 2020 comment from the Society for Maternal-Fetal Medicine on the management of intrahepatic cholestasis of pregnancy, which supports the use of ursodeoxycholic acid.^21^

Thus, clear evidence for the benefit of ursodeoxycholic acid in pregnancy is sparse, with a greater sample size required to achieve statistical power. Myometrium from women with intrahepatic cholestasis of pregnancy has greater oxytocin-mediated contractility than that from women without intrahepatic cholestasis of pregnancy,^22^ and, because cholic acid exposure increases the expression of oxytocin receptors in human myometrium,^23^ ursodeoxycholic acid-mediated alteration in the bile acid pool could reduce spontaneous preterm birth in women with intrahepatic cholestasis of pregnancy.^12^ There is experimental evidence that ursodeoxycholic acid treatment reduces the impact of pathological processes that are implicated in the causes of stillbirth in women with intrahepatic cholestasis of pregnancy, such as placental vasospasm^24^ and fetal arrhythmia (abnormal heart rate variability and the elevation of umbilical venous N-terminal pro-brain natriuretic peptide are associated with elevated maternal and fetal bile acid concentrations).^25^, ^26^ However, clinical trials powered to detect alterations in stillbirth rates would require participant numbers that are likely to be unfeasible given the disease prevalence.^27^

We therefore aimed to use data from existing literature to examine whether ursodeoxycholic acid affects adverse perinatal outcomes, predominantly stillbirth and preterm birth. We planned to use individual participant-level data to enable appropriate adjustment for the main confounders and the inclusion of observational studies, in addition to RCTs.

## Methods

### Search strategy and selection criteria

In this systematic review and individual participant data meta-analysis, we prospectively searched Ovid using the Map Term to Subject Heading feature to find Medical Subject Heading (MeSH) terms for inclusion in the subsequent literature searches. We then searched PubMed, Web of Science, Embase, MEDLINE, CINAHL, Global Health, MIDIRS, and Cochrane for articles on ursodeoxycholic acid use in women with intrahepatic cholestasis of pregnancy published between database inception, and Jan 1, 2020, using search terms referencing intrahepatic cholestasis of pregnancy, ursodeoxycholic acid, and perinatal outcomes, with MeSH additional search term permutations included in the search terms (appendix p 12). The reference lists of selected articles and relevant reviews were also searched to identify any manuscripts of potential relevance not already found in the database search. There were no language restrictions; publications that were not in English were translated by fluent speakers of the original language or Google Translate. Studies had to have ethical approval to share data and 30 or more study participants, and publications had to report at least one of: stillbirth, preterm birth, neonatal unit admission, meconium-stained amniotic fluid, or neonatal death (appendix p 12). Studies that did not report on any individual with intrahepatic cholestasis of pregnancy and bile acid concentrations of 40 μmol/L or more at any point in pregnancy were excluded because more adverse perinatal outcomes occur at these concentrations. Duplicates were removed. Relevant articles were selected by title and abstract and adherence to the inclusion and exclusion criteria and then full-text screening was done (appendix p 12); the searches and the selection were done in duplicate by JS and KP, and any disparities were arbitrated by CO. Two additional unpublished cohort studies—one from the UK and one from Italy—of women with intrahepatic cholestasis of pregnancy were included. The UK study included 254 women with intrahepatic cholestasis of pregnancy who had provided individual informed consent (study approved by the Ethics Committee of Hammersmith Hospital National Health Service Trust, London, UK [97/5197, 17/WA/0161, and 08/H0707/21]; Williamson C, unpublished). Details of the 85 women included in the Italian study are available online.^28^ To collect individual participant data, the corresponding authors of selected articles were contacted via email on at least two occasions; if no reply was received, at least one other author of the manuscript was contacted. The study protocol was pre-registered in PROSPERO, CRD42019131495.

### Data analysis

The data analysis plan was pre-specified (appendix p 3). Participating authors completed pseudo-anonymised spreadsheets reporting simple maternal demographics, intrahepatic cholestasis of pregnancy diagnostic and treatment details, and perinatal outcomes (appendix p 12). Participant details were provided with numbers that could only be de-identified by the supplying author, which enabled any data inaccuracies or questions to be asked of the original author but meant that the main dataset was anonymised for our analyses. Analyses were done in Stata, version 16.0. The primary outcome was stillbirth prevalence by ursodeoxycholic acid treatment in all studies. To compare women treated with ursodeoxycholic acid (at any dose and duration) with women who did not receive ursodeoxycholic acid, an individual participant data meta-analysis was done by use of multi-level mixed-effects logistic regression utilising the Stata function melogit, or logistic regression with a Huber–White correction when the mixed-effects regression did not converge, with participants nested within studies and (for multiple pregnancies) infants nested within mothers.^29^ Adjustment was done for bile acid concentrations at baseline, number of fetuses, and maternal parity, because of the established relationships between these confounders and adverse perinatal outcomes, and anticipated data availability.^3^, ^5^, ^30^, ^31^

Associations between bile acid concentrations and stillbirth (by ursodeoxycholic acid treatment) were compared, for singleton pregnancies from all studies, by use of the roccomp function in Stata. The effect of ursodeoxycholic acid treatment on this association was determined on the basis of peak bile acid concentrations during treatment and for the whole pregnancy. Baseline bile acid concentration was defined as the highest bile acid concentration before treatment randomisation (for RCTs) or at diagnosis (assuming that most women treated with ursodeoxycholic acid in observational studies started treatment rapidly after baseline). Women with bile acid concentrations recorded at the beginning of the study (baseline) and later in their pregnancy were included in the comparison of the timing of bile acid measurement and the association with stillbirth.

Secondary maternal (safety) outcomes (analysed according to the same methods as the primary outcome) were the onset of labour (spontaneous or induced, including elective caesarean), the mode of delivery (spontaneous vaginal, assisted vaginal, elective caesarean, or emergency caesarean), pre-eclampsia, gestational diabetes (not reported as the majority of women were diagnosed with intrahepatic cholestasis of pregnancy after screening for [and diagnosing] gestational diabetes), and post-partum haemorrhage. Modifications to the PROSPERO planned analyses are documented in the appendix (p 2). In response to the relatively small number of participants for whom data were available, we anticipated that the number of stillbirths reported would result in insufficient power to measure the effect of ursodeoxycholic acid; therefore, we modified our objectives to evaluate a secondary composite outcome (stillbirth or preterm birth). Additional perinatal secondary outcomes were: all components of the composite outcome (spontaneous birth, iatrogenic birth, and total preterm birth), early preterm birth (<34 gestational weeks), neonatal unit admission, meconium-stained amniotic fluid, umbilical cord arterial pH of less than 7·0, an Apgar score of less than 7 at 5 min of life, perinatal death, small for gestational age, large for gestational age, and spontaneous preterm birth. By use of a Cox's proportional hazards model, a prespecified survival analysis was done to measure the risk of spontaneous preterm birth (defined as birth following spontaneous labour onset before 37 gestational weeks) and, post-hoc, iatrogenic preterm birth (defined as clinician-initiated birth before 37 gestational weeks) over time for participants with singleton pregnancies in RCTs, stratified by ursodeoxycholic acid treatment. Participants were divided according to baseline bile acid concentrations into predefined categories and hazard ratios (HRs) were calculated that compared ursodeoxycholic acid treatment status and bile acid category. Post-hoc, we did a further analysis of the HRs of spontaneous preterm birth split by bile acid category (<40 μmol/L and ≥40 μmol/L).

Individual patient data analyses were done for all studies and in different subgroups, which were produced by limiting analyses to RCTs only, singleton pregnancies only, or two-arm studies only (appendix p 26). Results are presented as adjusted odds ratios (aOR), with 95% CIs, and p values are reported. p values less than 0·05 were considered significant. Missing data were handled by exclusion.

Logistic regression of subgroups was done to measure the effects of treatment by bile acid concentrations (<40 μmol/L, 40–99 μmol/L, and ≥100 μmol/L),^3^, ^5^ and, for the composite outcome, gestational age at diagnosis (<32 gestational weeks or ≥32 gestational weeks),^32^ and maximum daily ursodeoxycholic acid dose (<1 g *vs* ≥1 g; 1 g was the median value for the whole cohort). Interactions between groups were calculated by use of the likelihood ratio.

Given that we only received individual participant data from four of fourteen RCTs (822 [59·2%] of 1389 pregnancies; appendix pp 13–16), aggregate data from all published RCTs were compared in a post-hoc conventional fixed-effects meta-analysis, deriving summary effects by use of Mantel–Haenszel methods. Between-study heterogeneity was estimated by use of the χ^2^ test and calculation of *I*^2^. Funnel plots were produced to review potential publication bias. The number of study participants or studies with women with bile acid concentrations less than 40 μmol/L were not exclusion criteria; studies were otherwise selected on the basis of the original search strategy. Studies that reported clear randomisation in their design, and had at least one group who received ursodeoxycholic acid and another group who did not receive ursodeoxycholic acid, were used. We defined high-quality studies, for this purpose, as being those that were double-blinded, placebo-controlled, and had the intervention administered until delivery. Lower-quality studies were those not fulfilling all high-quality criteria. Aggregate data on stillbirth, spontaneous preterm birth, and overall preterm birth (defined as birth before 37 gestational weeks) were extracted from the original manuscripts in duplicate by CO and JS.

Study quality assessment tools from the National Heart, Lung, and Blood Institute were used to provide a quality score for each included publication.^33^ These scores were independently assessed by JS and NJW, with arbitration by CO. This study is registered in PROSPERO, CRD42019131495.

### Role of the funding source

The funders of the study had no role in study design, data collection, data analysis, data interpretation, or writing of the report.

## Results

**Figure 1:**
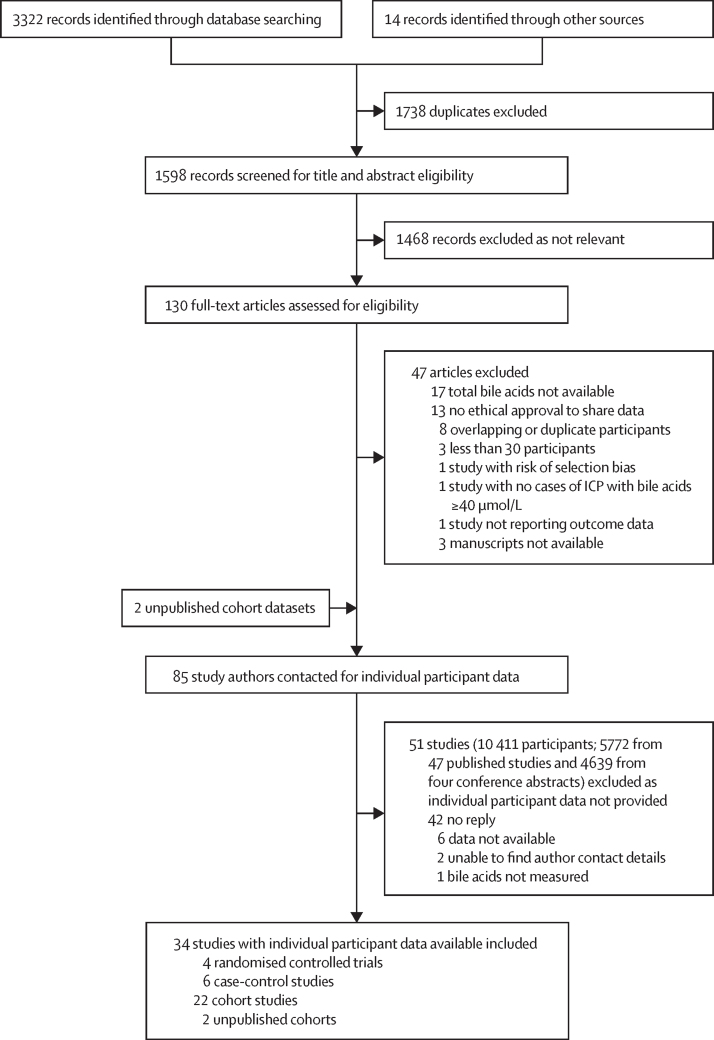
Study selection Adapted from PRISMA and PRISMA individual participant data. ICP=intrahepatic cholestasis of pregnancy. PRISMA=Preferred Reporting Items for Systematic Reviews and Meta-Analyses.

85 studies fulfilled our inclusion criteria (figure 1). Individual participant data were provided for 32 published studies (6670 pregnancies), including four RCTs; these data were enriched with data from two unpublished cohort studies (339 pregnancies; figure 1; appendix pp 13–17). Of the 7009 participants for whom data were provided, 35 were excluded from the analysis because they had an unknown treatment and 6974 had sufficient data for inclusion (822 from the four RCTs), of whom 4726 (67·8%) took ursodeoxycholic acid and 2248 (32·2%) did not take ursodeoxycholic acid (appendix p 18). Characteristics, including data quality scores, of the studies used in the meta-analysis can be found in the appendix (pp 13–16).

The prevalence of stillbirth did not differ between women treated with ursodeoxycholic acid (35 [0·7%] of 5097 fetuses) and women not treated with ursodeoxycholic acid (12 [0·6%] of 2038 fetuses; aOR 1·04, 95% CI 0·35–3·07; p=0·95; table 1); the prevalence of stillbirth also did not differ by ursodeoxycholic acid treatment when considering singleton pregnancies or RCTs alone (Table 1, Table 2), or when considering two-arm studies alone (appendix pp 20, 27). Women with peak bile acid concentrations of 100 μmol/L or more from both treatment groups had a higher prevalence of stillbirth (2·04% for those treated with ursodeoxycholic acid and 2·00% for those not treated with ursodeoxycholic acid) than did those with peak bile acid concentrations less than 100 μmol/L (0·47% for those treated with ursodeoxycholic acid and 0·37% for those not treated with ursodeoxycholic acid; appendix p 29). In women with singleton pregnancies, ursodeoxycholic acid treatment did not affect the association between peak bile acid concentration and stillbirth, whether the highest bile acid measurement for the whole pregnancy was used (p=0·69) or bile acid concentration measured after treatment initiation was used (p=0·72; appendix p 30). The highest bile acid concentration recorded throughout the whole pregnancy was the best predictor of stillbirth compared with other timepoints (at baseline and after baseline), but no difference was found between timings of bile acid measurement and the association of stillbirth with bile acid concentrations (p=0·15; appendix p 30)

**Table 1:** Perinatal and maternal outcomes according to ursodeoxycholic acid treatment using individual participant data from all studies

	**All studies (n=34)**				**Randomised controlled trials (n=4)**			
	Treated with ursodeoxycholic acid	Not treated with ursodeoxycholic acid	aOR (95% CI)	p value	Treated with ursodeoxycholic acid	Not treated with ursodeoxycholic acid	aOR (95% CI)	p value
**Perinatal outcomes**								
Stillbirth	35/5097 (0·7%)	12/2038 (0·6%)	1·04 (0·35–3·07)	p=0·95	1/439 (0·2%)	3/429 (0·7%)	0·29 (0·04–2·42)	p=0·25
Composite outcome	2480/5314 (46·7%)	514/2213 (23·2%)	1·28 (0·86–1·91)	p=0·22	75/439 (17·1%)	107/429 (24·9%)	0·60 (0·39–0·91)	p=0·016
Total preterm birth (<37 weeks' gestation)	2476/5287 (46·8%)	508/2208 (23·0%)	1·30 (0·87–1·94)	p=0·20	75/438 (17·1%)	106/428 (24·8%)	0·61 (0·40–0·92)	p=0·019
Spontaneous preterm birth (<37 weeks' gestation)	767/4871 (15·7%)	169/2175 (7·8%)	0·55 (0·35–0·88)	p=0·012	30/438 (6·8%)	52/428 (12·1%)	0·56 (0·31–1·01)	p=0·052
Iatrogenic preterm birth (<37 weeks' gestation)	1293/4871 (26·5%)	306/2175 (14·1%)	1·13 (0·75–1·70)	p=0·55	45/438 (10·3%)	54/428 (12·6%)	0·80 (0·48–1·33)	p=0·39
Meconium-stained amniotic fluid	703/4694 (15·0%)	304/1987 (15·3%)	0·69 (0·50–0·95)	p=0·022	55/436 (12·6%)	85/425 (20·0%)	0·51 (0·34–0·77)	p=0·001
Apgar score less than 7 at 5 min	156/5008 (3·1%)	37/2150 (1·7%)	1·09 (0·57–2·07)	p=0·80	10/437 (2·3%)	11/419 (2·6%)	0·85 (0·37–1·94)	p=0·70
Umbilical cord arterial pH less than 7·0	6/1649 (0·4%)	8/871 (0·9%)	0·86 (0·15–4·82)	p=0·86	3/164 (1·8%)	3/161 (1·9%)	0·71 (0·12–4·10)	p=0·70
Large for gestational age	492/4116 (12·0%)	220/1432 (15·4%)	1·57 (1·09–2·25)	p=0·014	65/402 (16·2%)	45/395 (11·4%)	1·51 (1·00–2·29)	p=0·052
Small for gestational age	351/4116 (8·5%)	83/1432 (5·8%)	0·98 (0·60–1·59)	p=0·92	23/402 (5·7%)	20/395 (5·1%)	1·25 (0·62–2·50)	p=0·53
Neonatal unit admission	1298/4787 (27·1%)	457/2081 (22·0%)	0·96 (0·70–1·32)	p=0·79	58/438 (13·2%)	78/427 (18·3%)	0·67 (0·43–1·03)	p=0·067
Perinatal death	34/3403 (1·0%)	9/1606 (0·6%)	1·37 (0·32–5·87)	p=0·67	1/378 (0·3%)	2/363 (0·6%)	0·40 (0·04–3·63)	p=0·41
**Maternal outcomes**								
Pre-eclampsia	206/3618 (5·7%)	121/1574 (7·7%)	1·14 (0·53–2·47)	p=0·74	1/51 (2·0%)	0/43 (0·0%)	NA	NA
Unassisted vaginal birth	1926/3842 (50·1%)	1146/1853 (61·8%)	1·08 (0·83–1·41)	p=0·58	261/412 (63·3%)	253/397 (63·7%)	0·94 (0·70–1·27)	p=0·70

Data are n/N (%), unless otherwise specified. ORs were calculated using logistic regression with Huber–White correction, with study level as a fixed effect and clustering by fetuses for those with multifetal pregnancies. For stillbirth, the composite outcome (stillbirth or preterm birth), preterm birth, and other perinatal outcomes, analyses were done by number of fetuses; for maternal outcomes, analyses were done by number of pregnancies. Data were adjusted by baseline bile acid concentration and maternal parity. aOR=adjusted odds ratio. NA=not applicable.

**Table 2:** Perinatal and maternal outcomes according to ursodeoxycholic acid treatment for singleton pregnancies using individual participant data from all studies

	**All studies (n=34)**				**Randomised controlled trials (n=4)**			
	Treated with ursodeoxycholic acid	Not treated with ursodeoxycholic acid	aOR (95% CI)	p value	Treated with ursodeoxycholic acid	Not treated with ursodeoxycholic acid	aOR (95% CI)	p value
**Perinatal outcomes**								
Stillbirth	21/3700 (0·6%)	11/1801 (0·6%)	0·71 (0·10–4·99)	p=0·73	1/388 (0·3%)	2/367 (0·5%)	0·40 (0·03–4·66)	p=0·46
Composite outcome	1262/3881 (32·5%)	353/1955 (18·1%)	0·68 (0·48–0·97)	p=0·034	42/388 (10·8%)	67/367 (18·3%)	0·51 (0·33–0·78)	p=0·002
Total preterm birth (<37 weeks' gestation)	1258/3855 (32·6%)	347/1952 (17·8%)	0·69 (0·48–0·98)	p=0·040	42/387 (10·9%)	66/366 (18·0%)	0·51 (0·33–0·79)	p=0·002
Spontaneous preterm birth (<37 weeks' gestation)	365/3702 (9·9%)	109/1932 (5·6%)	0·54 (0·31–0·94)	p=0·028	18/387 (4·7%)	32/366 (8·7%)	0·46 (0·25–0·86)	p=0·015
Iatrogenic preterm birth (<37 weeks' gestation)	740/3702 (20·0%)	218/1932 (11·3%)	0·88 (0·56–1·37)	p=0·56	24/387 (6·2%)	34/366 (9·3%)	0·63 (0·37–1·09)	p=0·10
Meconium-stained amniotic fluid	499/3360 (14·9%)	266/1760 (15·1%)	0·58 (0·41–0·83)	p=0·003	48/385 (12·5%)	72/365 (19·7%)	0·54 (0·36–0·81)	p=0·003
Apgar score <7 at 5 min	115/3607 (3·2%)	32/1902 (1·7%)	0·76 (0·31–1·88)	p=0·56	5/386 (1·3%)	7/361 (1·9%)	0·67 (0·21–2·15)	p=0·51
Umbilical cord arterial pH <7·0	3/1351 (0·2%)	8/779 (1·0%)	0·37 (0·04–3·30)	p=0·37	1/136 (0·7%)	3/141 (2·1%)	0·27 (0·02–2·89)	p=0·28
Large for gestational age	462/2878 (16·1%)	213/1217 (17·5%)	1·55 (1·07–2·25)	p=0·021	65/354 (18·4%)	44/338 (13·0%)	1·55 (1·02–2·37)	p=0·040
Small for gestational age	121/2878 (4·2%)	39/1217 (3·2%)	1·33 (0·61–2·93)	p=0·48	11/354 (3·1%)	8/338 (2·4%)	1·30 (0·51–3·28)	p=0·58
Neonatal unit admission	729/3407 (21·4%)	371/1842 (20·1%)	0·72 (0·50–1·06)	p=0·097	34/387 (8·8%)	48/366 (13·1%)	0·64 (0·40–1·02)	p=0·061
Perinatal death	19/2747 (0·7%)	9/1439 (0·6%)	0·54 (0·06–4·83)	p=0·58	1/335 (0·3%)	2/320 (0·6%)	0·40 (0·34–4·65)	p=0·46
**Maternal outcomes**								
Pre-eclampsia	122/2971 (4·1%)	99/1469 (6·7%)	0·75 (0·26–2·19)	p=0·60	1/48 (2·1%)	0/40 (0·0%)	NA	NA
Unassisted vaginal birth	1860/3224 (57·7%)	1121/1740 (64·4%)	1·06 (0·80–1·39)	p=0·70	255/387 (65·9%)	247/366 (67·5%)	0·92 (0·67–1·25)	p=0·60

Data are n/N (%), unless otherwise specified. ORs were calculated by use of multilevel mixed-effects logistic regression, with study level as a fixed effect and adjustment for baseline bile acid concentration and maternal parity. aOR=adjusted odds ratio. NA=not applicable.

Results for most secondary outcomes can be found in Table 1, Table 2. Adjusted analysis of the entire dataset revealed that ursodeoxycholic acid treatment was associated with a reduced risk of spontaneous preterm birth (table 1), in contrast to the unadjusted comparisons, in which this reduction was not evident (appendix p 28). This reduction was seen in RCTs when restricted to singleton pregnancies (table 2) and when restricted to singleton pregnancies in two-arm studies (appendix pp 20, 28). Ursodeoxycholic acid treatment was also associated with a reduced risk of total preterm birth for women with singleton and multifetal pregnancies in RCTs (table 1) and when considering only singleton pregnancies for all studies, adjusting for the main confounders (table 2). Ursodeoxycholic acid did not reduce the prevalence of early preterm birth (birth before 34 gestational weeks; appendix p 19). Heterogeneity at the study level was statistically significant for iatrogenic preterm birth when considering all studies (*I*^2^=97·44%; impact of study on the risk of iatrogenic preterm birth OR 2·31, 95% CI 1·10–4·82). Removal of single-arm studies from the analysis did not alter perinatal and maternal outcomes for singleton pregnancies, but did suggest a negative impact of ursodeoxycholic acid on preterm birth outcomes when multifetal pregnancies were also included (appendix p 20).

**Figure 2:**
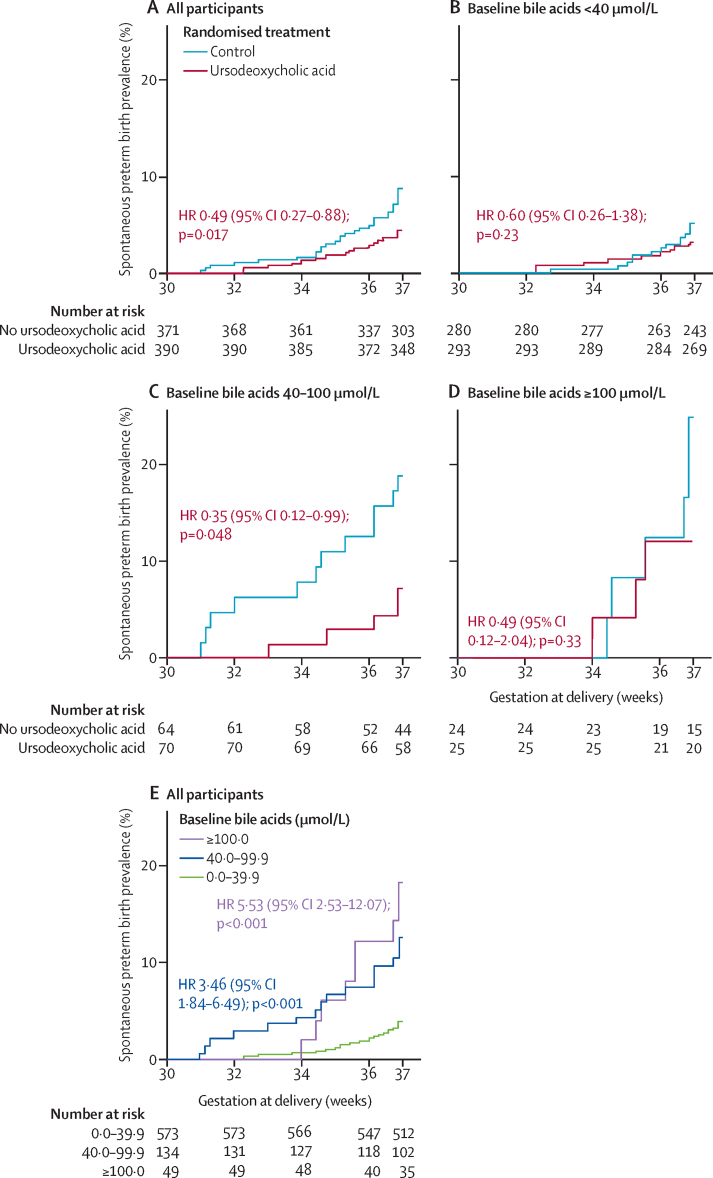
Kaplan–Meier plots of the prevalence of spontaneous preterm birth by gestational week of birth, according to ursodeoxycholic acid use and disease severity at randomisation Analyses were done by use of individual participant data from women with singleton pregnancies participating in randomised controlled trials. Kaplan–Meier plots for all women (A), women with baseline bile acid concentrations less than 40 μmol/L (B), women with baseline bile acid concentrations between 40 μmol/L and 100 μmol/L (C), and women with baseline bile acid concentrations of 100 μmol/L or more (D). HRs compare women randomly assigned to ursodeoxycholic acid treatment with those randomly assigned to placebo. (E) All women by baseline bile acid concentration; HRs compare women with baseline bile acid concentrations of 40·0–99·9 μmol/L and 100·0 μmol/L or more to those with baseline bile acid concentrations less than 40·0 μmol/L. HR=hazard ratio.

A survival analysis of women with singleton pregnancies in RCTs showed that the risk of spontaneous preterm birth was lower for women treated with ursodeoxycholic acid than for women not treated with ursodeoxycholic acid (figure 2A). When the impact of ursodeoxycholic acid treatment was compared in women with different peak bile acid concentrations at baseline, ursodeoxycholic acid treatment was significantly associated with a reduced risk of spontaneous preterm birth only in those with serum bile acid concentrations between 40 μmol/L and 100 μmol/L (figure 2B–D; appendix p 31), although interaction testing between the groups was not significant (p=0·67). Overall, peak bile acid concentration at baseline was associated with spontaneous preterm birth (figure 2E). The impact of ursodeoxycholic acid treatment on iatrogenic preterm birth was not significant (appendix p 32).

For RCTs alone, ursodeoxycholic acid significantly reduced the composite outcome, largely due to reduced total preterm birth, with the number needed to treat equalling 15 (95% CI 9–54; table 1). The reduction in total preterm birth was probably caused by a reduction in spontaneous preterm birth (table 1). We confirmed these findings for the composite outcome in participants with singleton pregnancies, with adjustment for baseline confounders (table 2), with the number needed to treat equalling 14 (95% CI 8–42). There was no difference in the prevalence of the composite outcome between groups with differing baseline bile acid concentrations, between women diagnosed before 32 gestational weeks and women diagnosed at 32 gestational weeks or after, or by the dose of ursodeoxycholic acid prescribed (appendix p 21).

Women treated with ursodeoxycholic acid had lower odds of meconium-stained amniotic fluid and higher odds of large-for-gestational age babies than did women not treated with ursodeoxycholic acid (table 1). There were no differences in the prevalences of neonatal unit admission, having an umbilical cord arterial pH of less than 7·0 or an Apgar score of less than 7 at 5 min, small-for-gestational-age babies, or perinatal death, between the group treated with ursodeoxycholic acid and the group not treated with ursodeoxycholic acid (table 1). There were significantly higher prevalences of neonatal unit admission and meconium-stained amniotic fluid for participants with baseline bile acid concentrations of 40 μmol/L or greater (compared with those with concentrations <40 μmol/L) and for participants with baseline bile acid concentrations of 100 μmol/L or greater (compared with those with concentrations <100 μmol/L; appendix p 22). Baseline bile acid concentrations of 100 μmol/L or more were associated with a higher prevalence of neonatal death compared with bile acid concentrations less than 100 μmol/L (appendix p 22).

There were no significant differences in maternal outcomes (eg, the induction of labour and post-partum haemorrhage [appendix p 23] and unassisted vaginal birth [Table 1, Table 2]) between women treated with ursodeoxycholic acid and women not treated with ursodeoxycholic acid, when considering all studies and when considering only RCTs. Treatment with ursodeoxycholic acid did not impact the prevalence of pre-eclampsia for the entire cohort; insufficient data were available on the development of pre-eclampsia in women participating in RCTs to perform a reliable analysis (table 1). Maternal outcomes were not related to bile acid concentrations (appendix p 24).

**Figure 3:**
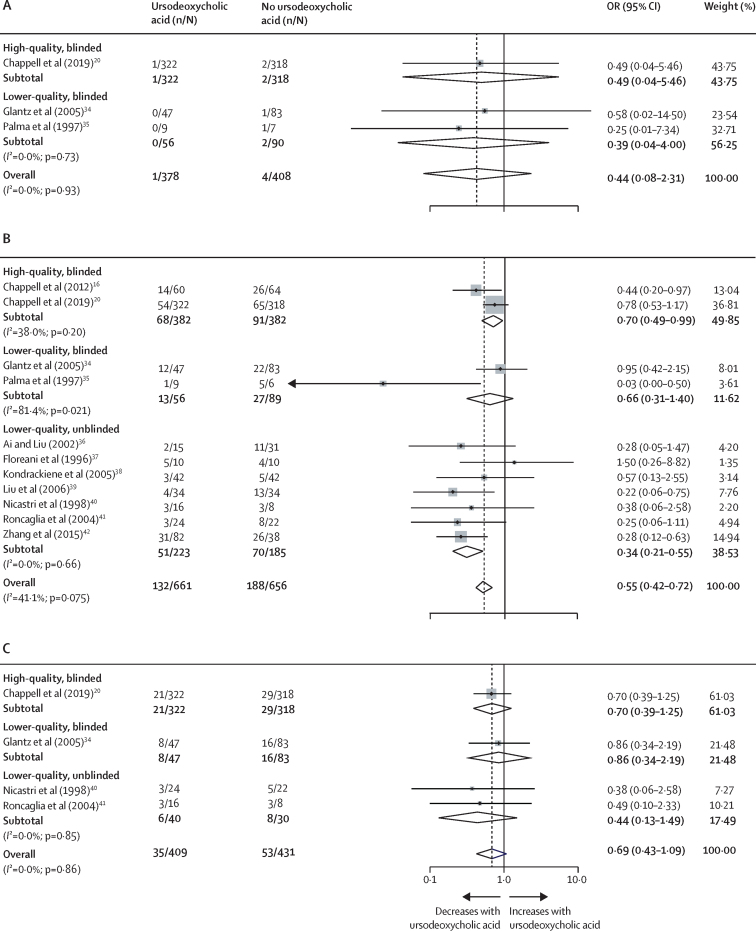
Stillbirth and preterm birth in women in RCTs of ursodeoxycholic acid treatment (A) Stillbirth. (B) All preterm births before 37 gestational weeks. (C) Spontaneous preterm births before 37 gestational weeks. Aggregate published data were used. Stillbirth was analysed by number of fetuses, except for Glantz and colleagues (in which stillbirth was analysed by number of pregnancies). The black diamonds are the individual study point estimates, the grey boxes reflect the weight of the individual study, the horizontal lines represent the CIs of the effect estimates, the white diamonds represent the pooled ORs and CIs, and the vertical dotted line represents the pooled OR. Study weight was calculated from the inverse variance. Preterm birth was analysed by number of fetuses, except for Glantz and colleagues, Kondrackiene and colleagues, Roncaglia and colleagues, and Palma and colleagues (in which preterm birth was analysed by number of pregnancies). OR=odds ratio.

Given the size of the effect of data adjustment on the observational studies versus the RCTs, and the limitation of having the individual participant data from only four RCTs, we decided to do a post-hoc aggregate data meta-analysis on published RCTs to compare the effect of ursodeoxycholic acid with the effect of any other treatment on outcomes. We identified 14 studies (appendix p 25) for inclusion. Ursodeoxycholic acid treatment did not affect the odds of stillbirth (figure 3A), but did reduce the odds of total preterm birth (p<0·001; figure 3B), although the reduction in spontaneous preterm birth specifically was not significant (p=0·11; figure 3C). Only two of the RCTs, in which ursodeoxycholic acid treatment was continued until delivery, were double-blinded, and, for these studies, the effect size for preterm birth reduction was less than that of the unblinded studies (figure 3B). For all comparisons, publication bias was not evident on funnel plots (appendix p 33).

## Discussion

Using an individual participant data meta-analysis, we showed that ursodeoxycholic acid treatment was associated with lower odds of a composite outcome of stillbirth and preterm birth, and total preterm births, in RCTs, and lower odds of spontaneous preterm birth in all studies (and when considering only singleton pregnancies); however, stillbirth rate did not differ between women treated with ursodeoxycholic acid and women not treated with ursodeoxycholic acid. Our findings were supported by an analysis of the aggregate data from published RCTs, which also showed that ursodeoxycholic acid treatment was associated with a reduced odds of total preterm births. Of the prespecified secondary maternal and perinatal outcomes, the odds of meconium-stained amniotic fluid reduced with ursodeoxycholic acid treatment.

Although ursodeoxycholic acid did not reduce the odds of early preterm birth before 34 gestational weeks, prevention of late preterm birth (before 37 gestational weeks) is of considerable benefit; these babies are at a higher risk of post-partum respiratory impairment, delayed feeding, early childhood mortality, neurodevelopmental disability, and longer-term cognitive defects than are children born at term.^43^ Another individual patient data meta-analysis showed that, for women with intrahepatic cholestasis of pregnancy and serum bile acid concentrations less than 100 μmol/L, stillbirth prevalence was no higher than that of the background population.^5^ This evidence means that, when interpreted by clinicians, the prevalence of iatrogenic preterm delivery for women with lower bile acid concentrations is likely to decrease,^44^ which could result in more fetuses that could benefit from the ability of ursodeoxycholic acid to reduce spontaneous preterm birth.

This meta-analysis has shown the value of well designed RCTs in intervention studies. When considering individual participant data from all study designs, adjustment of comparisons by the main confounders (baseline bile acid concentrations, parity, and number of fetuses) reversed the effect of ursodeoxycholic acid, reflecting how poorly matched the treatment groups were in terms of the main influencers of perinatal outcomes in intrahepatic cholestasis of pregnancy. Similarly, removal of single-arm studies from the analysis of perinatal and maternal outcomes did alter the effect of ursodeoxycholic acid on preterm birth outcomes when multifetal pregnancies were included (but not when singleton pregnancies were included). This finding implicates different mechanisms by which intrahepatic cholestasis of pregnancy affects preterm birth or stillbirth in multifetal pregnancies, consistent with a previously reported absence of association between bile acid concentration and stillbirth in multifetal pregnancies that contrasts to that between bile acid concentration and singleton pregnancies.^5^ Alternatively, this finding might show the impact of unmatched comparator groups, particularly when outcomes are analysed by number of fetuses, rather than by the number of pregnancies. The results of the aggregate meta-analysis are consistent with previous studies that have shown effect sizes to be overestimated in unconcealed or unblinded RCTs;^45^ only two of the RCTs, in which ursodeoxycholic acid treatment was continued until delivery, were double-blinded, and for these studies the effect size for preterm birth reduction was less than that of the unblinded studies. This overestimation of effect size might explain why historical studies have suggested additional perinatal benefits of ursodeoxycholic acid that have not been revealed in this meta-analysis (eg, on neonatal unit admission and Apgar scores).^15^ Differences between studies and the data collected limited the baseline adjustments that we were able to do and prevented use of an inverse probability treatment weighting approach; thus, results from the more comparable groups participating in RCTs are likely to be more reliable than are data from observational studies. The risk of selection bias for studies included in the independent participant data meta-analysis is a further limitation to the interpretation of our findings, which was mitigated in part by use of the aggregate data meta-analysis.

This study did not show a significant reduction in stillbirth with ursodeoxycholic acid treatment, despite attempting to include all data available. Stillbirth is a relatively rare outcome, and therefore the number of participants required to obtain sufficient power to detect any difference is likely to be restrictive. Limiting future studies to include women at greatest risk (eg, those with bile acid concentrations ≥100 μmol/L) would reduce the numbers needed to evaluate the effect of ursodeoxycholic acid on the risk of stillbirth, but might be unfeasible. Although we were able to include data from the largest RCT of ursodeoxycholic acid treatment,^20^ data were not available for many other studies, which limited the sample size available for comparison. Clinicians cannot, therefore, reassure women that treatment with ursodeoxycholic acid reduces the risk of stillbirth. Similarly, comprehensive data on ursodeoxycholic acid treatment duration and dose escalation were not available for this study, barring the provision of specific prescribing guidance for clinicians. Differences in laboratory methods for measuring bile acid concentration and bile acid reference ranges between centres also complicate interpretation.

To our knowledge, we are the first to report a reduction of spontaneous preterm birth in singleton pregnancies with ursodeoxycholic acid treatment. This finding was not evident from the aggregate data meta-analysis, due to limited reporting of this outcome with a standardised definition and incomplete reporting of this outcome by number of fetuses. A Cochrane review also did not show this reduction in spontaneous preterm birth with ursodeoxycholic acid, probably because of incomplete reporting in all studies and separate comparisons of ursodeoxycholic acid by comparator group (eg, placebo and S-adenosyl-methionine).^14^ We compared ursodeoxycholic acid treatment with any other treatment on the basis of the scarcity of evidence for perinatal benefit for other treatments.^46^ Similarly, by combining independent participant data from multiple studies, we were able to identify treatment benefits not seen in the largest RCT of ursodeoxycholic acid.^20^ For policy makers, it is reassuring that the 2019 RCT of ursodeoxycholic acid treatment in women with intrahepatic cholestasis of pregnancy did not find a difference in the total cost for ursodeoxycholic acid treatment compared with placebo (adjusted cost difference per patient −£429 [95% CI −1235 to 377]; adjusted p=0·30), or in reported adverse events.^20^ However, we did not show that ursodeoxycholic acid treatment improved all adverse perinatal outcomes, and it is clear that ursodeoxycholic acid cannot prevent all cholestasis-related adverse perinatal effects. Similarly, ursodeoxycholic acid has little benefit for maternal pruritus,^14^ and an effective alternative treatment is currently lacking. Thus, there is a clear need for complementary treatments for gestational cholestasis.

In summary, this meta-analysis suggests that ursodeoxycholic acid treatment in women with intrahepatic cholestasis of pregnancy reduces the risk of preterm birth. Previous work has shown that there is an increased risk of preterm birth in women with peak bile acid concentrations of 40 μmol/L or more (compared with women with peak bile acid concentrations <40 μmol/L);^5^ ursodeoxycholic acid treatment should therefore be considered for these women with disease onset before 37 weeks' gestation.

## Data sharing

Summary data will be available with publication (with no end date) upon reasonable request to Catherine Williamson (catherine.williamson@kcl.ac.uk), subject to an appropriate data sharing agreement. The study protocol and statistical analysis plan will be available in the appendix with publication of this Article. Individual participant data from the unpublished UK cohort will be available with publication of this Article, unless consent for data sharing was withheld by the participant, upon reasonable request to Catherine Williamson (catherine.williamson@kcl.ac.uk), subject to an appropriate data sharing agreement. Unpublished data from the Italian cohort for the purpose of meta-analysis is available online.^28^ Other individual participant data should be requested from the original study authors.

## Declaration of interests

CO and H-UM are consultants for Mirum Pharmaceuticals. CW is a consultant for Mirum Pharmaceuticals and GlaxoSmithKline. KK is an unpaid consultant for Myriad Pharmaceuticals. WMH reports non-financial support from the Falk Foundation, during the conduct of the study, and is co-author of the Cochrane review on pharmacological interventions for treating intrahepatic cholestasis of pregnancy.^14^ RMT reports grants from Tommy's and the Lauren Page Trust during the conduct of the study. All other authors declare no competing interests.

## References

[1] Bicocca MJ, Sperling JD, Chauhan SP (2018). Intrahepatic cholestasis of pregnancy: review of six national and regional guidelines. Eur J Obstet Gynecol Reprod Biol.

[2] Ovadia C, Williamson C (2016). Intrahepatic cholestasis of pregnancy: recent advances. Clin Dermatol.

[3] Glantz A, Marschall HU, Mattsson LA (2004). Intrahepatic cholestasis of pregnancy: relationships between bile acid levels and fetal complication rates. Hepatology.

[4] Herrera CA, Manuck TA, Stoddard GJ (2018). Perinatal outcomes associated with intrahepatic cholestasis of pregnancy. J Matern Fetal Neonatal Med.

[5] Ovadia C, Seed PT, Sklavounos A (2019). Association of adverse perinatal outcomes of intrahepatic cholestasis of pregnancy with biochemical markers: results of aggregate and individual patient data meta-analyses. Lancet.

[6] Kawakita T, Parikh LI, Ramsey PS (2015). Predictors of adverse neonatal outcomes in intrahepatic cholestasis of pregnancy. Am J Obstet Gynecol.

[7] Li Q, Dutta A, Kresge C, Bugde A, Feranchak AP (2018). Bile acids stimulate cholangiocyte fluid secretion by activation of transmembrane member 16A Cl^−^ channels. Hepatology.

[8] Beuers U, Hohenester S, de Buy Wenniger LJ, Kremer AE, Jansen PL, Elferink RP (2010). The biliary HCO(3)(-) umbrella: a unifying hypothesis on pathogenetic and therapeutic aspects of fibrosing cholangiopathies. Hepatology.

[9] Pusl T, Vennegeerts T, Wimmer R, Denk GU, Beuers U, Rust C (2008). Tauroursodeoxycholic acid reduces bile acid-induced apoptosis by modulation of AP-1. Biochem Biophys Res Commun.

[10] Wan JF, Chu SF, Zhou X (2018). Ursodeoxycholic acid protects interstitial Cajal-like cells in the gallbladder from undergoing apoptosis by inhibiting TNF-α expression. Acta Pharmacol Sin.

[11] Estiú MC, Monte MJ, Rivas L (2015). Effect of ursodeoxycholic acid treatment on the altered progesterone and bile acid homeostasis in the mother-placenta-foetus trio during cholestasis of pregnancy. Br J Clin Pharmacol.

[12] Manna LB, Ovadia C, Lövgren-Sandblom A (2019). Enzymatic quantification of total serum bile acids as a monitoring strategy for women with intrahepatic cholestasis of pregnancy receiving ursodeoxycholic acid treatment: a cohort study. BJOG.

[13] Tribe RM, Dann AT, Kenyon AP, Seed P, Shennan AH, Mallet A (2010). Longitudinal profiles of 15 serum bile acids in patients with intrahepatic cholestasis of pregnancy. Am J Gastroenterol.

[14] Walker KF, Chappell LC, Hague WM, Middleton P, Thornton JG (2020). Pharmacological interventions for treating intrahepatic cholestasis of pregnancy. Cochrane Database Syst Rev.

[15] Kong X, Kong Y, Zhang F, Wang T, Yan J (2016). Evaluating the effectiveness and safety of ursodeoxycholic acid in treatment of intrahepatic cholestasis of pregnancy: a meta-analysis (a prisma-compliant study). Medicine (Baltimore).

[16] Chappell LC, Gurung V, Seed PT, Chambers J, Williamson C, Thornton JG (2012). Ursodeoxycholic acid versus placebo, and early term delivery versus expectant management, in women with intrahepatic cholestasis of pregnancy: semifactorial randomised clinical trial. BMJ.

[17] Grand'Maison S, Durand M, Mahone M (2014). The effects of ursodeoxycholic acid treatment for intrahepatic cholestasis of pregnancy on maternal and fetal outcomes: a meta-analysis including non-randomized studies. J Obstet Gynaecol Can.

[18] Bacq Y, Sentilhes L, Reyes HB (2012). Efficacy of ursodeoxycholic acid in treating intrahepatic cholestasis of pregnancy: a meta-analysis. Gastroenterology.

[19] Shen Y, Zhou J, Zhang S (2019). Is it necessary to perform the pharmacological interventions for intrahepatic cholestasis of pregnancy? A Bayesian network meta-analysis. Clin Drug Investig.

[20] Chappell LC, Bell JL, Smith A (2019). Ursodeoxycholic acid versus placebo in women with intrahepatic cholestasis of pregnancy (PITCHES): a randomised controlled trial. Lancet.

[21] Lee RH, Greenberg M, Metz TD, Pettker CM (2021). Society for Maternal-Fetal Medicine consult series #53: intrahepatic cholestasis of pregnancy. Am J Obstet Gynecol.

[22] Israel EJ, Guzman ML, Campos GA (1986). Maximal response to oxytocin of the isolated myometrium from pregnant patients with intrahepatic cholestasis. Acta Obstet Gynecol Scand.

[23] Germain AM, Kato S, Carvajal JA, Valenzuela GJ, Valdes GL, Glasinovic JC (2003). Bile acids increase response and expression of human myometrial oxytocin receptor. Am J Obstet Gynecol.

[24] Lofthouse EM, Torrens C, Manousopoulou A (2019). Ursodeoxycholic acid inhibits uptake and vasoconstrictor effects of taurocholate in human placenta. FASEB J.

[25] Miragoli M, Kadir SH, Sheppard MN (2011). A protective antiarrhythmic role of ursodeoxycholic acid in an in vitro rat model of the cholestatic fetal heart. Hepatology.

[26] Vasavan T, Deepak S, Jayawardane IA (2020). Fetal cardiac dysfunction in intrahepatic cholestasis of pregnancy is associated with elevated serum bile acid concentrations. J Hepatol.

[27] Flenady V, Gardener G, Boyle FM (2019). My baby's movements: a stepped wedge cluster randomised controlled trial to raise maternal awareness of fetal movements during pregnancy study protocol. BMC Pregnancy Childbirth.

[28] Indraccolo U, Catagini S, Bianchi B, Morano D (March, 2020). Intrahepatic cholestasis of pregnancy—database Arcispedale Sant'Anna of Ferrara. https://www.researchgate.net/publication/340175016_Intrahepatic_cholestasis_of_pregnancy_-_Database_Arcispedale_Sant'Anna_of_Ferrara#fullTextFileContent.

[29] Rabe-Hesketh S, Skrondal A (2012). Multilevel and longitudinal modelling using Stata.

[30] Visintin C, Mugglestone MA, James D, Kilby MD (2011). Antenatal care for twin and triplet pregnancies: summary of NICE guidance. BMJ.

[31] Stillbirth Collaborative Research Network Writing Group (2011). Association between stillbirth and risk factors known at pregnancy confirmation. JAMA.

[32] Estiú MC, Frailuna MA, Otero C (2017). Relationship between early onset severe intrahepatic cholestasis of pregnancy and higher risk of meconium-stained fluid. PLoS One.

[33] National, Heart, Lung, and Blood Institute Quality assessment tool for observational cohort and cross-sectional studies. https://www.nhlbi.nih.gov/health-topics/study-quality-assessment-tools.

[43] Petrou S (2019). Health economic aspects of late preterm and early term birth. Semin Fetal Neonatal Med.

[44] Palmer KR, Xiaohua L, Mol BW (2019). Management of intrahepatic cholestasis in pregnancy. Lancet.

[45] Schulz KF, Chalmers I, Hayes RJ, Altman DG (1995). Empirical evidence of bias. Dimensions of methodological quality associated with estimates of treatment effects in controlled trials. JAMA.

[46] Gurung V, Middleton P, Milan SJ, Hague W, Thornton JG (2013). Interventions for treating cholestasis in pregnancy. Cochrane Database Syst Rev.

