# Supplementary material for: Ursodeoxycholic acid in intrahepatic cholestasis of pregnancy: a systematic review and individual participant data meta-analysis
Source: Lancet Gastroenterol Hepatol. 2021 Apr 27;6(7):547–58. doi: 10.1016/S2468-1253(21)00074-1 (PMC8192305; doi:10.1016/S2468-1253(21)00074-1)
Supplement: Supplementary appendix [file mmc1.pdf]

THE LANCET
Gastroenterology & Hepatology

## Supplementary appendix

This appendix formed part of the original submission and has been peer reviewed. We post it as supplied by the authors.

Supplement to: Ovadia C, Sajous J, Seed PT, et al. Ursodeoxycholic acid in intrahepatic cholestasis of pregnancy: a systematic review and individual participant data meta-analysis. *Lancet Gastroenterol Hepatol* 2021; published online April 26. https://doi.org/10.1016/S2468-1253(21)00074-1.

# Appendix

Modifications to PROSPERO incorporated into the Data Analysis Plan……………………………………. 2

Data Analysis Plan……………………………………………………………………………… 3

Deviations from the Data Analysis Plan………………………………………………………… 11

Table S1 – Search terms used for identification of relevant literature……………………………….. 12

Table S2 – Inclusion and exclusion criteria for selection of studies……………………………….. 12

Table S3 – Data requested from authors………………………………………………………… 12

Table S4 – Description of studies used for individual participant data meta-analysis………………………... 13

Table S5 – Studies selected but not included in the individual participant data meta-analysis………………. 17

Table S6 – Characteristics of women providing individual participant data, from all studies (Group A)…… 18

Table S7 – Early preterm birth rate according to ursodeoxycholic acid treatment using individual participant data, from all studies (Groups A, B, E, F)………………………………………………………… 19

Table S8 – Perinatal and maternal outcomes according to ursodeoxycholic acid treatment, using individual participant data, excluding single-arm studies (Groups C, D)………………………………………. 20

Table S9 – Impact of bile acid concentration at baseline, gestation at diagnosis, and maximum daily ursodeoxycholic acid dose on the composite outcome using individual participant data, from randomised controlled trials (Group E)…………………………………………………………....21

Table S10 – Impact of bile acid concentration at baseline on perinatal outcomes using individual participant data, from all studies (Group A)……………………………………….………………………. 22

Table S11 – Maternal outcomes according to ursodeoxycholic acid treatment using individual participant data, from all studies (Groups A, E)……………………………………….……………………… 23

Table S12 – Impact of bile acid concentration at baseline on maternal pregnancy outcomes using individual participant data, from all studies (Group A)……………………………………………..…….. 24

Table S13 – Randomised controlled trials included in the aggregate data meta-analysis (Group G)..………..25

Figure S1 – Schematic of dataset subgroups …………………...……………………………………..26

Figure S2 – Rates of stillbirth and preterm birth for women receiving or not receiving ursodeoxycholic acid during their pregnancies, using individual participant data from all two-arm studies (Group C)……27

Figure S3 – Proportion and incidence of stillbirths and total number of singleton pregnancies by peak total bile acid concentration and ursodeoxycholic acid treatment using individual participant data, from all studies (Group B)………………………………………………………………..…………... 29

Figure S4 – Receiver operating characteristic curves to determine the association between stillbirth and peak bile acid concentration for women with singleton pregnancies, using individual participant data, from all studies (Group B)…………...………………………………………………………………. 30

Figure S5 – Kaplan-Meier survival plots of spontaneous preterm birth rate by gestational week of birth, according to ursodeoxycholic acid use and disease severity at randomisation for women with singleton pregnancies, using individual participant data from randomised controlled trials (Group F)……… 31

Figure S6 – Kaplan-Meier survival plots of iatrogenic preterm birth rate by gestational week of birth, according to ursodeoxycholic acid use and disease severity at randomisation for women with singleton pregnancies, using individual participant data, from randomised controlled trials (Group F)…….…32

Figure S7 – Funnel plots of studies in the aggregate data meta-analysis of randomised controlled trials…….33

Supplementary References………………………………………………………………………… 34

**Modifications to PROSPERO incorporated into the Data Analysis Plan**

The Data Analysis Plan was written following completion of the systematic review, based upon the numbers of participants anticipated to be eligible for analysis and an informal summary of data points available from early study responders, but prior to data review or analysis.

1. Addition of the composite outcome of stillbirth and preterm birth as a secondary outcome. During data collection, it became apparent that sufficient data on stillbirth would not be available to provide power to determine whether UDCA had an effect on its rate. We therefore added analysis of preterm birth (including overall and spontaneous preterm birth, with rates <37/40 gestation and <34/40 gestation) to include a new composite (stillbirth and overall preterm birth).
2. Maternal pre-eclampsia and gestational diabetes mellitus. Meta-regression by diagnostic criteria. Information on the diagnostic criteria for both conditions were rarely reported in eligible studies, therefore meta-regression by diagnostic criteria was not attempted.
3. Subgroup analysis of the composite and main secondary outcomes was planned by important confounders (baseline bile acid concentration, gestation of disease onset, and UDCA dose).
4. Additional secondary outcomes were included, such as labour onset, post-partum haemorrhage rate and perinatal death. Results for unassisted vaginal birth were presented instead of caesarean section rate and large for gestational age/ small for gestational age rates rather than birthweight centile as we believed that these were more clinically-relevant outcomes.

## Data Analysis Plan

**The impact of ursodeoxycholic acid on perinatal outcomes in cholestatic pregnancies – an Individual Participant Data Meta-analysis**

**Study name**

Individual participant data meta-analysis to determine the effects of ursodeoxycholic acid treatment for intrahepatic cholestasis of pregnancy

**Study registration (Prospero)**

PROSPERO 2019 CRD42019131495
https://www.crd.york.ac.uk/prospero/display_record.php?RecordID=131495

**Funders**

NIHR Senior Investigator Award

**Sign off**

Professor Catherine Williamson Date 10/3/2020

Contents

Study name ... 3
Study registration ... 3
Authors ... 3
Funders ... 3
Sign off ... 3
Abbreviations ... 6
Definitions ... 6
Version history ... 6
1. Hypothesis,  aims and objectives ... 7
Hypothesis ... 7
Aims ... 7
Objectives ... 7
2. Inclusion and exclusion criteria ... 7
Study inclusion criteria ... 7
Study exclusion criteria ... 7
Participant inclusion criteria ... 7
Participant exclusion criteria ... 7
3. Data to be collected ... 8
Study-level data ... 8
Essential information needed from all studies ... 8
Maternal demographic and antenatal predictors ... 8
Neonatal and perinatal details ... 8
Additional information to be collected where available ... 8
Maternal demographic and antenatal predictors ... 8
Neonatal and perinatal details ... 8
4. Analysis and presentation of results ... 8
Data search procedure and results ... 9
5. Primary outcome ... 9
Secondary analyses of primary outcome ... 9
(1) By study quality ... 9
(2) By bile acid level at diagnosis ... 9
(3) By gestation at diagnosis ... 9
(4) By maximum prescribed UDCA dosing ... 9
(5) By total exposure to UDCA ... 9
6. Management of women with ICP ... 9
7. Maternal outcomes ... 10
8. Secondary outcomes ... 10
Composite outcome ... 10
All components of composite ... 10

Spontaneous preterm birth ........ 10
Other individual outcomes ........ 10
9. Additional analyses........ 10
S1 Sensitivity analysis 1: excluding unpublished data ........ 10
S2 Sensitivity analysis 2: per-protocol analysis excluding women on treatments other than UDCA ........ 10
S3 Include as a confounder the background stillbirth rate for each country of study, maternal age, BMI ........ 10
S4 Subgroup of women with pre-pregnancy liver conditions ........ 10
S5 Subgroup of women with other pre-pregnancy conditions ........ 10
S6 Subgroup of women with other pregnancy-induced conditions ........ 10

**Abbreviations:**

| | |
|---|---|
| ICP | intrahepatic cholestasis of pregnancy (obstetric cholestasis) |
| IPD | individual participant data |
| TBA | total bile acids |
| UDCA | ursodeoxycholic acid |
| NNU | neonatal unit |
| MSAF | meconium-stained amniotic fluid |
| SVD | spontaneous (unassisted) vaginal delivery |
| AVD | assisted vaginal delivery |
| ELCS | elective caesarean section |
| EMCS | emergency caesarean section |
| SGA | small for gestational age |
| LGA | large for gestational age |
| PPH | postpartum haemorrhage |
| PP | postpartum |

**Definitions:**
Intrahepatic cholestasis of pregnancy (ICP): defined according to local diagnostic criteria.

Severe intrahepatic cholestasis of pregnancy: Bile acid concentration ≥40μmol/L in pregnancy with associated pruritus, with all signs and symptoms resolving postnatally.

Small for gestational age (SGA): birthweight ≤$10^{th}$ centile.

Large for gestational age (LGA): birthweight ≥$90^{th}$ centile.

Postpartum haemorrhage (PPH): post-delivery maternal blood loss >500ml.

Gestational diabetes mellitus (GDM): diagnosed according to local diagnostic criteria.

**Version history**
First draft (rough notes written by CO) 7 Feb 2020

Version 0·1 (7 Feb 2020) Written by PTS in collaboration with CO, using the first draft as a guideline

Version 0·2 (26 Feb 2020) Minor amendments by CW

Version 0·3 (26 Feb 2020) Extended and clarified by PTS, CO & JS

Sections and tables given consistent numbering, secondary analysis of primary outcome clarified, time to SPTB added, references added. Extension & clarification of bile acid data to be collected.

Version 0·4 (5 March 2020) Further work by PTS, CO & CW

New section 6 added on management of women with ICP. New subgroup analysis by total exposure.

Version 0·5 (9 March 2020) Changes approved by JS, current study progress updated. Author list and proof-reading completed by CO

Version 1·0 (10 March 2020) Version sign-off by CW

Version 1·1 (5 October 2020) Formatting for publication by CO

## 1. Hypothesis, Aims and objectives

### Hypothesis

That UDCA treatment in severe intrahepatic cholestasis of pregnancy is associated with reduced rates of adverse fetal outcomes.

### Aims

To improve the management of women with severe and non-severe ICP by a better understanding of the effects of UDCA on maternal and neonatal outcomes. Our main interest is in stillbirth, but, as this is rare, we will also investigate a composite outcome that is more likely to be adequately powered.

### Objectives

*Primary objective:* To determine whether women with ICP taking UDCA have lower rates of stillbirth than untreated women with ICP.

*Secondary objectives:*

1. To determine whether women with ICP taking UDCA have lower rates of preterm birth (iatrogenic and spontaneous), meconium-stained amniotic fluid, neonatal unit admission, low Apgar score, low umbilical artery pH, birthweight centile and perinatal death.
2. To determine whether women with ICP taking UDCA have lower rates of a composite adverse perinatal outcome (stillbirth and preterm birth) than untreated women with ICP.
3. To determine whether women treated with UDCA have altered rates of adverse maternal outcomes (pre-eclampsia, gestational diabetes mellitus, postpartum haemorrhage).

## 2. Inclusion and exclusion criteria

### Study inclusion criteria

- Prospective case-control studies, prospective cohort studies, retrospective cohort studies, population-based studies, and randomised controlled trials that reported bile acid levels and neonatal outcomes were included. Personal communication of unpublished cohorts
- ICP diagnosis based upon pruritus and elevated serum bile acid levels
- Reporting of bile acid concentrations, UDCA usage and perinatal outcomes
- Ethical approval to share the data

### Study exclusion criteria

- Case reports, studies not comprising cohorts or successive cases seen in a unit
- <30 study participants
- Diagnosis of ICP not using serum bile acid levels
- Studies that did not report the serum bile acid level or any perinatal outcomes

### Participant inclusion criteria

- Women with ICP diagnosed by pruritus in pregnancy and raised serum bile acids, not attributed to an alternative underlying cause of liver pathology
- Study participants with data recorded for ***either*** peak TBA pre-treatment/pre-randomisation/at diagnosis and peak TBA post-treatment/post placebo, ***or*** peak TBA during pregnancy if pre-treatment is not available or treatment not administered
- Study participant with one or more of the following antenatal predictors and perinatal details recorded: parity; number of fetuses; UDCA treatment; live birth/stillbirth; gestation at delivery; neonatal death; meconium presence/absence

### Participant exclusion criteria

- Studies investigating only mild disease (bile acids <40µmol/L)

## 3. Data to be collected

### Study-level data

Numbers of treated and untreated, ICP diagnostic criteria, country, outcomes, study quality, dates.

### Essential information needed from all studies

Maternal demographic and antenatal predictors:

- Parity
- Number of fetuses
- UDCA treatment (yes/no)
- *Either*:
    - Peak TBA pre-treatment/pre-randomisation/at diagnosis
    - Peak TBA post-treatment/post placebo/post diagnosis
- *Or*:
    - Peak TBA during pregnancy if pre-treatment is not available

Neonatal and perinatal details (Where relevant, information collected for each neonate):

- Gestation at delivery, stillbirth

### Additional information to be collected where available

Maternal demographic and antenatal predictors:

- Age, BMI, gestation at ICP diagnosis, pre-eclampsia, gestational diabetes, pre-existing diabetes, other liver conditions, chronic hypertension, metformin treatment, insulin treatment
- UDCA treatment (timing and dosage), cholestyramine treatment, rifampicin treatment, S-adenosyl methionine treatment, vitamin K treatment
- Maximum total bile acids after treatment with UDCA/placebo/other disease modifying drugs

Neonatal and perinatal details (Where relevant, information collected for each neonate):

- Mode of delivery, induction of labour status, blood loss, birthweight, birthweight centile, sex, umbilical cord artery pH, Apgar at 5 minutes, meconium-stained amniotic fluid, neonatal unit admission, neonatal death
- Preterm birth before 37 weeks, operative delivery, blood loss ≥ 500 mL, SGA (< $10^{th}$ Intergrow birthweight centile), umbilical artery pH < 7, Apgar ≤ 7 (5 minutes, meconium-stained amniotic fluid, neonatal unit admission, neonatal death

## 4. Analysis and presentation of results

The meta-analysis of results will investigate three things: the size and direction of effect, consistency between studies, strength of evidence. We are most interested in whether the efficacy of UDCA varies with bile acid levels pre-treatment in 3 groups: <40 µmol/L, 40-99 µmol/L, 100+ µmol/L.

Because we have individual participant data, we are not restricted to standard meta-analysis techniques, but can use multi-level modelling, with participants nested within studies, and (for multiple pregnancy) infants within mothers (Rabe-Hesketh & Skrondal, 2012)[1]. Our main results will be for all studies together, with adjustment for possible confounding by parity and number of fetuses.

Sensitivity analyses (if there are sufficient data) will

1. Consider RCTs separately
2. Exclude low-quality studies (as defined by NHLBI study scoring system, 2020)[2]
3. Adjust for maximum bile acids pre-treatment/diagnosis (where available)
4. Adjust for maximum bile acid measurement

Data analysis will be in the statistical package Stata version 16·0 or later (StataCorp, College Station, Texas).

**Data search procedure and results**
Standard PRISMA flow diagram with numbers of studies and participants included and excluded with reasons

**5. Primary outcome**
Stillbirth rates by UDCA (any vs none). The analysis will be adjusted for bile acid level at diagnosis, prior to the start of UDCA, parity and number of fetuses.

Odds ratios are calculated using multilevel models, with fetus, mother and study considered as separate levels. The analysis will be adjusted for bile acid level at diagnosis, prior to the start of UDCA, parity, and number of fetuses.

The composite outcome is defined as any of stillbirth, iatrogenic or spontaneous preterm birth. It is recorded once for each fetus.

**Secondary analyses of primary outcome:**
(1) By study quality
Look at randomised studies only; check for heterogeneity between studies.

(2) By bile acid level at diagnosis (>100, over 100)
A subgroup analysis to work out the treatment effect in each subgroup. We will carry out an interaction test for moderation of effect.

(3) By gestation at diagnosis (<32weeks vs 32+)
A subgroup analysis to work out the treatment effect in each subgroup. We will carry out an interaction test for moderation of effect. Other groupings may be considered depending on the data.

(4) By maximum prescribed UDCA dosing
A dose-response analysis. This will include tests for differences in effect between the two dosage groups. Other groupings may be considered depending on the data.

The main comparison will be between <1 g vs ≥1 g maximum dose/day. Other major doses will be considered depending on the data.

(5) By total exposure to UDCA (in women whose pregnancy continued for at least 1 week after start of UDCA)
This analysis is designed to show the cumulative effect of UDCA treatment. There are technical problems: UDCA treatment duration and total exposure are closely linked to length of pregnancy and hence to stillbirth and exact UDCA treatment schedule is not generally collected. To some extent they are addressed by limiting the analysis to women who were on UDCA long enough for there to be an effect. Groups will be defined when the data is available.

Odds ratios are calculated using multilevel models, with fetus, mother and study considered as separate levels. Adjustment is made for bile acid level at diagnosis, parity and number of fetuses.

The composite outcome is defined as any of stillbirth, iatrogenic or spontaneous preterm birth.

**6. Management of women with ICP**
This analysis will attempt to identify the level of TBA corresponding to an unacceptable risk separately according to UDCA treatment; as in our previous paper (Ovadia 2019).[3] UDCA groups may be changed depending on numbers available.

Risk of stillbirth by maximum level of TBA (separately for women ever or never prescribed UDCA during the pregnancy) and ROC curves of UDCA as a predictor of stillbirth will be presented.

**7. Maternal (safety) outcomes**
Maternal outcomes: onset of labour as spontaneous, induced, or elective caesarean; mode of delivery as spontaneous vaginal, assisted vaginal, elective caesarean, emergency caesarean; pre-eclampsia; gestational diabetes mellitus; postpartum haemorrhage; postpartum blood loss.

Pruritus specifically not included, as no standardisable scale exists.

Odds ratios are calculated using multilevel models, with fetus, mother and study considered as separate levels. Adjustment is made for parity and number of fetuses.

Stillborn babies are not included in onset of labour or mode of delivery analyses.

**8. Secondary outcomes**
To be analysed according the same methods as the primary outcome

Composite outcome (stillbirth and preterm birth)

All components of composite (including data from studies without the full composite outcome)

Spontaneous Preterm delivery
This will be based on multilevel parametric survival analysis of all women using time from diagnosis to spontaneous preterm delivery, with censoring of non-cases at 37 weeks, or (for iatrogenic delivery) at delivery. As with the QUiPP app (Watson 2017),[4] we will develop a suitable parametric model, using Akaike and Bayesian Information Criteria (AIC & BIC) to determine the best model. Predictors to be considered will include study (random effect), UDCA (yes/no), bile acid level at diagnosis, parity, and gestation at diagnosis.

Other individual outcomes
Secondary outcomes to analyse: NNU admission; MSAF; umbilical arterial pH <7·0; Apgar <7 at 5 minutes of life; neonatal death; perinatal death; SGA; LGA.

**9. Additional analyses**
S1 Sensitivity analysis 1: excluding unpublished data
Restricted to primary outcome, composite and most important safety features: pre-eclampsia, GDM

S2 Sensitivity analysis 2: per-protocol analysis excluding women on treatments other than UDCA
Exclude women prescribed rifampicin, cholestyramine, S-adenosyl methionine (SAMe); data on dexamethasone prescription not available.

Other possible subgroup analyses (depending on availability of data):

S3 Include as a confounder the background stillbirth rate for each country of study, maternal age, BMI

S4 Subgroup of women with pre-pregnancy liver conditions

S5 Subgroup of women with other pre-pregnancy conditions
For example, diabetes Type 1 or 2, CHT, CKD

S6 Subgroup of women with other pregnancy-induced conditions
For example, gestational diabetes, preeclampsia. Ideally, pre-diagnosis only, but we probably will not know when they were diagnosed

**Deviations from the Data Analysis Plan**

1. Use of multilevel modelling. Particularly for perinatal outcomes, multilevel modelling failed to converge. We therefore used logistic regression with Huber-White correction for clustering by mother/fetus, and study level as a fixed effect in the majority of analyses (method used indicated in the relevant table footnote).
2. Analysis for singleton pregnancies only. *Post hoc*, we elected to further investigate the main study outcomes for singleton pregnancies only, results for which are presented in the supplementary material.
3. Section 4. Sensitivity analysis excluding low quality studies. We did not specifically perform this sensitivity analysis as considerations to study quality were included within the analysis: using IPD enabled us to adjust for the main confounders, and the analysis of aggregate data from RCTs was divided by masking, and the comparator treatment used, as a reflection of study quality; heterogeneity between studies was studied. The effect of adjustment on primary outcomes for the observational cohorts was so marked that we elected to limit the subanalyses by major disease factors (bile acids/ gestation of disease onset / UDCA dosing) to the RCTs only.
4. Section 5. Analysis by total exposure to UDCA – as anticipated in the Data Analysis Plan, availability of this was limited, therefore this analysis was not performed.
5. Section 6. Given that previous studies demonstrated the association between bile acid concentrations and stillbirth occurred for women with singleton pregnancies, we elected, *post hoc*, to perform this analysis restricted to singleton pregnancies.
6. Section 7. Maternal outcomes. Rates of gestational diabetes mellitus (GDM) were collected and analysed, however the gestation of onset of GDM occurred prior to the onset of ICP for the majority of women. Assuming that the majority of women were diagnosed with GDM at around 28/40, from the whole cohort there were only 44 women diagnosed with ICP before this gestation and then GDM, of whom 39/251 (15·5%) were taking UDCA and 5/20 (25%) not taking UDCA. We felt these numbers were not sufficient to reliably perform this comparison. Similarly, there was not enough data on rates of pre-eclampsia from participants within RCTs to perform a reliable comparison of the effect of UDCA.
7. Section 8. Secondary outcomes – Spontaneous preterm birth. Given the proportion of iatrogenic preterm births within the cohort, we elected to perform this analysis with different methodology, presenting hazard ratios of preterm birth by disease category according to baseline bile acid concentration. Additionally, rates of neonatal death reported were too low to be reliable in analysis, thus results for this comparison are not presented.
8. Section 9. Sensitivity analyses. Due to very small numbers of affected women, sensitivity analyses are not presented for the exclusion of unpublished cohorts, women given additional disease-modifying treatments and pre-pregnancy conditions. As numbers of stillbirths were relatively low and confidence intervals wide, analysis by background stillbirth rates of the countries was not performed.

**Table S1. Search terms used for identification of relevant literature**

| **Search terms** |
|---|
| cholestasis, intrahepatic, of pregnancy; pregnancy-related cholestasis; recurrent intrahepatic cholestasis of pregnancy; obstetric cholestasis; cholestasis, intrahepatic of pregnancy; cholestasis, pregnancy-related; familial intrahepatic cholestasis of pregnancy; intrahepatic cholestasis of pregnancy |
| pregnancy outcome; fetal outcome; fetal distress; obstetric labour complication*; obstetric labor complication*; pregnancy, high*risk; delivery, obstetric; labour, obstetric; labor, obstetric; live birth; obstetric labour, premature; obstetric labor, premature; premature birth; cesarean section; caesarean section; abortion, spontaneous; stillbirth; fetal death; infant mortality; maternal mortality; perinatal mortality; gestational age; infant, low birth weight; Apgar score; pregnancy outcome*; pregnancy complication; obstetric outcome*; obstetric complication*; normal birth*; live birth*; premature birth*; preterm birth*; preterm deliver*; born preterm; cesarean*; c-section; LSCS; caesarean*; miscarriage*; stillbirth*; intrauterine death*; neonatal death*; postpartum haemorrhage*; postpartum hemorrhage*; postpartum complication*; special care baby unit admission*; SCBU admission*; neonatal intensive care unit admission*; NICU admission*; neonatal unit*; NNU admission*; small for gestational age; SGA; intra-uterine growth restriction; IUGR; Apgar; foetal distress; foetal outcome; foetal death; pregnancy loss; fetal demise; foetal demise |

**Table S2. Inclusion and exclusion criteria for selection of studies**

| | |
|---|---|
| **Inclusion criteria** | Prospective case-control studies, prospective cohort studies, retrospective cohort studies, population-based studies and randomised controlled trials. ICP was defined as pruritus in pregnancy with elevated serum bile acid concentration, with or without elevated aminotransferase concentration. Severe ICP was diagnosed as above, but with bile acid concentration ≥40 μmol at any point in pregnancy. Where possible, participants were also offered an abdominal ultrasound scan to exclude the presence of gallstones, sludge, or other hepatic pathologies, and serum screening to confirm postpartum resolution. The minimum study sample size was 30 participants. Publications had to report at least one of stillbirth, preterm birth, neonatal unit admission, meconium-stained amniotic fluid, or neonatal death. |
| **Exclusion criteria** | Studies with fewer than 30 participants, case reports, studies without cohorts or successive cases seen in a unit, or studies with a high risk of bias were excluded. Studies with no cases of severe disease (bile acids ≥ 40μmol/L) were excluded. Systematic reviews, meta-analyses and non-clinical studies were excluded although reference lists were checked for studies suitable for inclusion |

**Table S3. Data requested from authors**

| | |
|---|---|
| Maternal details | Parity, number of fetuses, gestation of delivery, induction of delivery, mode of delivery, estimated blood loss |
| ICP details | Gestation of diagnosis, bile acids at baseline, gestation of baseline, peak bile acids post baseline, gestation of peak bile acids post baseline |
| UDCA treatment | Gestation of commencement, treatment duration, maximum dose, maximum frequency |
| Other ICP treatments | SAMe, rifampicin, cholestyramine, vitamin K – use and gestations of use |
| Other conditions | Liver conditions, chronic hypertension, pre-eclampsia, pre-existing diabetes mellitus, gestational diabetes mellitus, metformin treatment, insulin treatment |
| Neonatal details | Birthweight, sex, abnormal cardiotocography, umbilical arterial pH, Apgar score at 5 minutes, meconium-staining of the amniotic fluid, neonatal unit admission, stillbirth, neonatal death |

**Table S4. Description of studies used for individual participant data meta-analysis**

| Author and year | Country | Data collection period | Number of participants (UDCA / no UDCA) | Design | ICP definition | Bile acid measurement | Data quality[2] | Major predictors | Outcome measures |
|---|---|---|---|---|---|---|---|---|---|
| Alessandrelli et al., 2009[5] | Italy | 2001-2007 | 84/70 | Retrospective cohort | Pruritus, ↑TBA | Fasting | 9 | Pruritus severity, TBA, individual BA | Apgar score, IUFD, PTB |
| Bacq et al., 2017[6] | France | 1999-2013 | 98/0 | Retrospective cohort | Gestational pruritus, ↑TBA or ALT on two occasions, no other liver disorder, or dermatoses of pregnancy | Fasting | 11 | Pruritus severity, TBA, ALT, AST, bilirubin, GGT, ALP, ABCB4 mutations, PT | GA delivery, IOL, MOD |
| Batsry et al., 2019[7] | Israel | 2011-2016 | 159/83 | Retrospective cohort | Pruritus, ↑TBA and/or ↑ALT or AST, absence of other diseases that may cause similar symptoms and laboratory abnormalities | Fasting | 11 | TBA, AST, ALT | Apgar score, BW, GA delivery, GDM, IOL, ICH, LGA, MOD, MSAF, NNU, PET, PND, RDS, SGA, UC arterial pH |
| Broom and Kane, 2016[8] | Australia | 2012-2014 | 116/39 | Retrospective cohort | Pruritus, ↑TBA and/or ↑ALT or AST with no other cause | Fasting and non-fasting | 8 | TBA | GA delivery, IOL, MSAF, NRHRM, PPH |
| Brouwers et al., 2015[9] | Netherlands | 2005-2012 | 151/63 | Retrospective cohort | Gestational pruritus, ↑TBA | Non-fasting | 12 | TBA, ALT, AST, bilirubin, GGT, ALP, LDH | Apgar score, BW, EBL, GA delivery, IOL, MOD, MSAF, NNU, PND, SGA |
| Brun-Furrer et al., 2016[10] | Switzerland | 2004-2014 | 345/0 | Retrospective case-control | Database coding, no other liver dysfunction | Non-fasting | 11 | TBA | Apgar score, BW, EBL, GA delivery, Hb, IOL, IUFD, MOD, MSAF, NNU, PND, UC arterial pH |
| Casagrandi et al., 2017[11] | UK | 2015-2016 | 56/27 | Retrospective cohort | ↑TBA | Non-fasting | 9 | TBA | IOL, MOD, MSAF, NNU |
| Castaño et al., 2006[12] | Argentina | 2004-2005 | 42/0 | Prospective case-control | Pruritus, ↑TBA, and ↑ALT/AST after 20/40, no other autoimmune disease, moderate-severe alcohol intake, HIV infection, Hepatitis A, B, or C infection, skin disease, or biliary obstruction, post-partum normalisation | Fasting | 8 | Pruritus severity, TBA, individual BA, ALT, AST, bilirubin, GGT, ALP | Apgar score, BW, GA delivery, IOL, MOD, MSAF, PND, SGA |
| Chappell et al., 2012[13] | UK | 2008-2011 | 56/55 | Randomised controlled trial | Gestational pruritus, ↑TBA or ALT, no other cause of pruritus or liver dysfunction (except concurrent hepatitis C or cholelithiasis) | Non-fasting | 12 | Pruritus severity, TBA, ALT, AST, bilirubin, GGT | Apgar score, BW, EBL, GA delivery, IOL, MOD, NNU, UC arterial pH |
| Chappell et al., 2019[14] | UK | 2015-2018 | 305/300 | Randomised controlled trial | Pruritus of no other cause, ↑TBA | Non-fasting | 13 | Pruritus severity, TBA, ALT, AST, bilirubin, GGT | Apgar score, BW, EBL, GA delivery, GDM, IOL, MOD, MSAF, NNU, PND, PTB, UC arterial pH |

| Author and year | Country | Data collection period | Number of participants (UDCA / no UDCA) | Design | ICP definition | Bile acid measurement | Data quality[2] | Major predictors | Outcome measures |
|---|---|---|---|---|---|---|---|---|---|
| Cui et al., 2018[15] | China | 2012-2015 | 37/0 | Prospective case-control | As per RCOG Guideline 2nd edition: i.e. gestational pruritus, ↑TBA and/or ↑ALT/AST, postpartum resolution, no other cause of itching or liver dysfunction | Fasting | 10 | TBA, individual BA, ALT, AST, bilirubin, GGT, ALP | BW, EBL, GA delivery, MOD, MSAF, PROM |
| Estiú et al., 2017[16] | Argentina | 2009-2013 | 298/84 | Prospective cohort | Pruritus, ↑TBA, no other cause for pruritus or hepatic disease | Fasting | 12 | TBA | Apgar score, GA delivery, IUFD, MSAF, NNU, PND, SGA, UC arterial pH |
| Gardiner et al., 2019[17] | Australia | 2014-2016 | 192/158 | Retrospective cohort | Pruritus with no other explanation, ↑TBA or ALT/AST, | Fasting | 8 | TBA, ALT, AST, bilirubin | GA delivery, IUFD, MOD, NNU |
| Geenes et al., 2014[18] | UK | 2010-2011 | 510/192 | Prospective population cohort | Pruritus without a rash, severe ICP: TBA≥ 40μmol/L | Non-fasting | 11 | TBA, ALT, AST, bilirubin, GGT | Apgar score, BW, GA delivery, IUFD, MOD, MSAF, NNU |
| Grymowicz et al., 2016[19] | Poland | 2005-2006 | 129/28 | Prospective case-control | Pruritus onset after 20/40, ↑TBA or ALT/AST, no other cause of pruritus, liver disease, or dermatological disease | Fasting | 10 | Pruritus severity, TBA, ALT, AST, bilirubin | GA delivery, MOD, UC arterial pH, Apgar score, MSAF, BW, NNU, ICH, RDS, IUFD |
| Günaydin et al., 2017[20] | Turkey | 2015 | 27/11 | Retrospective cohort | ICD codes, ↑TBA | Fasting | 9 | ↑TBA, ALT, AST, bilirubin, GGT, LDH, coagulation profile | MOD, GA delivery, IUFD, BW, Apgar score |
| Juusela et al., 2019[21] | USA | 2013-2017 | 49/18 | Retrospective cohort | ↑TBA, no other cause for liver disease | Fasting and non-fasting | 10 | TBA, AST | IUFD, MSAF NND, NNU, SPTB |
| Kebapcilar et al., 2010[22] | Turkey | 2008-2009 | 40/0 | Prospective case-control | Pruritus onset after 20/40, ↑TBA, no other liver disease or skin disease | Fasting | 9 | TBA, ALT, AST, ALP, lipid, and coagulation profiles | GA delivery, Apgar score |
| Kenyon et al., 2001[23] | UK | 1999-2001 | 29/44 | Prospective cohort | Pruritus, ↑TBA or ↑ALT/AST/GGT, no other liver disease, postnatal resolution | Non-fasting | 9 | Pruritus | MOD, APH, PPH, BW, NNU, IOL, IUFD, PND |
| Kohari et al., 2017[24] | USA | 2005-2014 | 355/516 | Retrospective cohort | Pruritus, ↑TBA | Non-fasting | 9 | TBA, ALT, AST | MSAF, IUFD, SGA, MOD, GA delivery, BW, Apgar score, UC arterial pH, NNU, RDS |
| Kondrackiene et al., 2005[25] | Lithuania | 1999-2002 | 33/27 | Randomised controlled trial | Pruritus starting in the 2nd/3rd trimesters, ↑ TBA, ALT, and/or AST in the absence of chronic liver disease, hepatic viral infections, skin disease, allergic disease, and symptomatic cholelithiasis | Fasting | 9 | Pruritus severity, TBA, and individual BA, ALT, AST | GA delivery, Apgar score, MOD, BW, IUFD |
| Lee et al., 2007[26] | USA | 2000-2007 | 37/80 | Retrospective cohort | ICP notes coding, pruritus, ↑TBA, no rash | Non-fasting | 12 | TBA, ALT, AST, bilirubin | MSAF, NRHRM, PPH, NNU, IUFD |

| Author and year | Country | Data collection period | Number of participants (UDCA / no UDCA) | Design | ICP definition | Bile acid measurement | Data quality[2] | Major predictors | Outcome measures |
|---|---|---|---|---|---|---|---|---|---|
| Liu et al., 2016[27] | China | 2006-2014 | 113/16 | Retrospective population cohort | Pruritus, ↑TBA | Fasting | 9 | TBA | MSAF, GA delivery, PET, GDM, IOL, Apgar score, NND, BW, LGA, SGA, RDS, NNU, IUFD |
| Madazli et al., 2015[28] | Turkey | 2003-2013 | 89/0 | Retrospective cohort | Pruritus, ↑TBA in the absence of any other liver or skin pathology | Fasting | 12 | TBA, ALT, AST, bilirubin, ALP | Apgar score, NRHRM, BW, GA delivery, MSAF, RDS, MOD, IUFD, NND, SGA |
| Majewska et al., 2019[29] | Poland | 2015-2017 | 57/0 | Retrospective cohort | Pruritus, ↑TBA, ↑LFTs | Fasting | 9 | TBA, ALT, AST, bilirubin | GA delivery, MOD, BW, SGA, LGA, Apgar score |
| Marathe et al., 2017[30] | Australia | 2001-2010 | 210/149 | Retrospective cohort | Pruritus, ↑TBA | Non-fasting | 11 | TBA | GDM, PET, GA delivery, Apgar score, UC arterial pH, BW, MSAF, RDS, NNU |
| Öztekin et al., 2009[31] | Turkey | 2004-2008 | 187/0 | Retrospective cohort | Pruritus, ↑TBA in the absence of other liver diseases, skin diseases, allergic disorders, symptomatic cholelithiasis, and ongoing viral infections affecting the liver | Fasting | 8 | TBA, ALT, AST, ALP, HDL, LDL, total cholesterol, triacylglycerol | Apgar score, GA delivery, IUFD, NND |
| Roncaglia et al., 2004[32] | Italy | 1996-2001 | 24/22 | Randomised controlled trial | Pruritus starting in the 2nd/3rd trimester in the absence of other pruritic medical conditions, ↑TBA, ALT and/or AST | Fasting | 11 | TBA, ALT, AST, bilirubin | MSAF, SGA, GA delivery, NNU, RDS, EBL, MOD, Apgar score, BW, UC arterial pH |
| Rook et al., 2012[33] | USA | 2005-2009 | 29/71 | Retrospective cohort | ICD9 coding, pruritus, onset in 2nd /3rd trimesters, no other cause for pruritus, chronic liver disease, acute fatty liver of pregnancy, or HELLP syndrome | Non-fasting | 9 | TBA, individual BA, ALT, AST, bilirubin, ALP, alb | MOD, IOL, GA delivery, BW, Apgar score, RDS, MSAF, NRHRM |
| Shan et al., 2016[34] | China | 2013-2015 | 310/12 | Retrospective cohort | ICD 10 coding, pruritus, ↑TBA, ALT, AST, no other cause of liver dysfunction, pruritus, gallstones, cholecystitis, or cirrhosis | Fasting | 12 | TBA, ALT, AST, LDH | PPH, MOD, MSAF, IUFD, GA delivery, BW, Apgar score, NNU, RDS, SGA |
| Turro et al., 2020[35] | Australia, Argentina, Sweden, UK | 2012-2017 | 217/36 | Retrospective cohort | Gestational pruritus, ↑TBA, no other cause for pruritus or hepatic dysfunction | Non-fasting | n/a* | Pruritus severity, TBA, ALT, AST, bilirubin | Apgar score, BW, EBL, GA delivery, GDM, IOL, IUFD, MOD, MSAF, NND, NNU, NRHRM, PTB, SPTB, UC arterial pH |
| Wong et al., 2009[36] | Ireland | 2004-2006 | 87/63 | Retrospective case-control | Pruritus, ↑TBA ± LFTs, no other cause for raised TBA | Fasting | 9 | TBA | MOD, IOL, Apgar score, NNU, NRHRM, PPH, BW, GA delivery |

| Author and year | Country | Data collection period | Number of participants (UDCA / no UDCA) | Design | ICP definition | Bile acid measurement | Data quality[2] | Major predictors | Outcome measures |
|---|---|---|---|---|---|---|---|---|---|
| *Unpublished cohorts at the time of analysis* | | | | | | | | | |
| Indraccolo et al. | Italy | 2016-2019 | 73/12 | Retrospective cohort | Pruritus, ↑TBA, no other liver disease | Fasting | n/a | TBA | Apgar score, BW, EBL, GA delivery, IOL, IPTB, MOD, MSAF, NND, NNU, NRHRM, IUFD, SPTB, UC arterial pH |
| Williamson et al. | UK | 2001-2019 | 182/72 | Prospective cohort | Gestational pruritus, ↑TBA, no other cause for pruritus or hepatic dysfunction | Non-fasting | n/a | Pruritus severity, TBA, ALT, AST, bilirubin | Apgar score, BW, EBL, GA delivery, GDM, IOL, IUFD, MOD, MSAF, NND, NNU, NRHRM, PTB, SPTB, UC arterial pH |

*Manuscript published after data inclusion, description in manuscript insufficient for quality assessment according to NHLBI cohort tool. Only women with ICP were included where case-control studies included women with uncomplicated pregnancies as the comparator group. alb: albumin, ALP: alkaline phosphatase, ALT: alanine aminotransferase, APH: antepartum haemorrhage, AST: aspartate aminotransferase, BW: birthweight, EBL: estimated blood loss, GA delivery: gestational age of delivery, GDM: gestational diabetes mellitus, GGT: Gamma-glutamyl transferase, Hb: haemoglobin, ICD: International Classification of Disease, ICH: intra-cerebral haemorrhage, IOL: induction of labour, IPTB: iatrogenic preterm birth, IUFD: intrauterine fetal death, LDH: lactate dehydrogenase, LFTs: liver function tests, LGA: large for gestational age, MOD: mode of delivery, MSAF: meconium-staining of the amniotic fluid, n/a: not applicable, NND: neonatal death, NNU: neonatal unit admission, NRHRM: non-reassuring heart rate monitoring, PET: pre-eclampsia, PPH: post-partum haemorrhage, PTB: preterm birth, RDS: respiratory distress syndrome, SGA: small for gestational age, SPTB: spontaneous preterm birth, TBA: serum total bile acids, UC: umbilical cord

**Table S5. Studies selected but not included in the individual participant data meta-analysis**

| Authors | Date | Country | Number of participants with ICP | Study type | Reason not included |
|---|---|---|---|---|---|
| Aksan Desteli et al.[37] | 2016 | Turkey | 113 | Retrospective case-control | No reply |
| Al-Obaidly et al.[38] | 2019 | Qatar | 163* | Population-based cohort | No reply |
| Al Shobaili et al.[39] | 2011 | Saudi Arabia | 76 | Prospective cohort | No reply |
| Alsulyman et al.[40] | 1996 | USA | 79 | Retrospective cohort | No reply |
| Binder et al.[41] | 2006 | Czech Republic | 78 | Randomised controlled trial | Data not available |
| Bolukbas el al.[42] | 2017 | Turkey | 59 | Retrospective cohort | No reply |
| Carballu-Núñez et al.[43] | 2015 | Spain | 71 | Retrospective cohort | No reply |
| Çelik et al.[44] | 2019 | Turkey | 370 | Retrospective cohort | No reply |
| Chen et al.[45] | 2013 | China | 106 | Retrospective cohort | No reply |
| Demir et al.[46] | 2014 | Turkey | 61 | Retrospective cohort | No reply |
| Erkenekli et al.[47] | 2015 | Turkey | 103 | Retrospective cohort | No reply |
| Feng et al.[48] | 2018 | China | 142 | Retrospective cohort | No reply |
| Frezza et al.[49] | 1990 | Italy | 30 | Randomised controlled trial | No reply |
| Friberg et al.[50] | 2016 | Denmark | 113 | Retrospective cohort | Data not available |
| Garcia-Flores et al.[51] | 2015 | Spain | 47 | Prospective cohort | No reply |
| Ge et al.[52] | 2016 | China | 196 | Prospective cohort | No reply |
| Glantz et al.[53] | 2005 | Sweden | 130 | Randomised controlled trial | Data not available |
| Heinonen et al.[54] | 1999 | Finland | 91 | Prospective cohort | Data not available |
| Herrera et al.[55] | 2018 | USA | 487 | Retrospective cohort | No reply |
| Jain et al.[56] | 2013 | India | 69 | Randomised controlled trial | No bile acid measurements |
| Jin et al.[57] | 2015 | China | 371 | Retrospective cohort | No reply |
| Joutsiniemi et al.[58] | 2015 | Finland | 103 | Retrospective cohort | No reply |
| Kawakita et al.[59] | 2015 | USA | 233 | Retrospective cohort | No reply |
| Koroglu et al.[60] | 2017 | Turkey | 40 | Prospective case-control | No reply |
| Kowalska-Kańka et al.[61] | 2013 | Poland | 41 | Prospective case-control | No reply |
| Labbe et al.[62] | 2018 | France | 138 | Retrospective cohort | No reply |
| Li et al.[63] | 2017 | China | 313 | Retrospective cohort | No reply |
| Lin et al.[64] | 2017 | China | 407 | Retrospective cohort | No reply |
| Liu et al.[65] | 2006 | China | 68 | Randomised controlled trial | No reply |
| Lu et al.[66] | 2014 | China | 88 | Retrospective cohort | No reply |
| Mahey et al.[67] | 2009 | India | 50* | Prospective case-control | No reply |
| Oztas et al.[68] | 2015 | Turkey | 117 | Prospective case-control | No reply |
| Nicastri et al.[69] | 1998 | Italy | 32 | Randomised controlled trial | No reply |
| Pata et al.[70] | 2011 | Turkey | 32 | Retrospective cohort | No reply |
| Proehl et al.[71] | 2017 | USA | 4329* | Retrospective cohort | No reply |
| Raddatz et al.[72] | 2019 | Germany | 97* | Retrospective cohort | No reply |
| Reyihanguli et al.[73] | 2017 | China | 207 | Prospective cohort | No contact details |
| Riikonen et al.[74] | 2000 | Finland | 48 | Randomised controlled trial | Data not available |
| Rioseco et al.[75] | 1994 | Chile, USA | 320 | Retrospective case-control | No contact details |
| Sargin Oruç et al.[76] | 2014 | Turkey | 57 | Prospective case-control | No reply |
| Singla et al.[77] | 2016 | India | 50 | Prospective case-control | No reply |
| Temel Yuksel et al.[78] | 2019 | Turkey | 40 | Prospective case-control | No reply |
| Turkmen et al.[79] | 2016 | Turkey | 37 | Retrospective case-control | No reply |
| Vural Yilmaz et al.[80] | 2017 | Turkey | 90 | Prospective case-control | No reply |
| Yayla Abide et al.[81] | 2017 | Turkey | 84 | Retrospective case-control | No reply |
| Zapata et al.[82] | 2005 | Chile | 48 | Retrospective cohort | No reply |
| Zecca et al.[83] | 2006 | Italy | 77 | Retrospective cohort | Data not available |
| Zhang et al.[84] | 2010 | China | 120 | Retrospective case-control | No reply |
| Zhang et al.[85] | 2014 | China | 40 | Retrospective cohort | No reply |
| Zhang et al.[86] | 2015 | China | 120 | Randomised controlled trial | No reply |
| Zhou et al.[87] | 2014 | China | 30 | Prospective case-control | No reply |

*denotes conference abstracts, all other studies were published full manuscripts

**Table S6. Characteristics of women providing individual participant data, from all studies (Group A)**

| | UDCA n (%)<br>(Total = 4726) | No UDCA n (%)<br>(Total = 2248) |
|---|---|---|
| **Baseline bile acid concentration (µmol/L)** | *N=3377* | *N=522* |
| <20·0 | 978 (29·0) | 216 (41·4) |
| 20·0-39·9 | 925 (27·4) | 172 (33·0) |
| 40·0-59·9 | 578 (17·1) | 58 (11·1) |
| 60·0-79·9 | 345 (10·2) | 28 (5·4) |
| 80·0-99·9 | 183 (5·4) | 14 (2·7) |
| 100·0-119·9 | 117 (3·5) | 12 (2·3) |
| ≥120 | 251 (7·4) | 22 (4·2) |
| **Peak bile acid concentration (µmol/L)** | *N=4576* | *N=2103* |
| <20·0 | 1148 (25·1) | 690 (32·8) |
| 20·0-39·9 | 1251 (27·3) | 558 (26·5) |
| 40·0-59·9 | 781 (17·1) | 352 (16·7) |
| 60·0-79·9 | 480 (10·5) | 216 (10·3) |
| 80·0-99·9 | 286 (6·3) | 96 (4·6) |
| 100·0-119·9 | 167 (3·6) | 57 (2·7) |
| ≥120 | 463 (10·1) | 134 (6·4) |
| **Nulliparity** | *N=4609* | *N=2161* |
| | 1565 (34·0) | 879 (40·7) |
| **Multifetal pregnancy** | *N=4722* | *N=2246* |
| | 795 (16·8) | 152 (6·8) |

UDCA: ursodeoxycholic acid

**Table S7. Early preterm birth rate according to ursodeoxycholic acid treatment using individual participant data, from all studies (Groups A, B, E, F – Appendix p26)**

| Outcome | All studies IPD | | | Randomised Controlled Trials IPD | | |
|---|---|---|---|---|---|---|
| | UDCA n/N (%) | No UDCA n/N (%) | aOR (95% CI, p value) | UDCA (n/N) (%) | No UDCA (n/N) (%) | aOR (95% CI, p value) |
| ***All pregnancies*** | | | | | | |
| Preterm birth <34 weeks' gestation | 436/5287 (8·2) | 77/2208 (3·5) | 0·89 (0·51-1·55, p=0·68) | 10/438 (2·3) | 24/428 (5·6) | 0·41 (0·16-1·06, p=0·066) |
| Spontaneous preterm birth <34 weeks' gestation | 182/4871 (3·7) | 39/2175 (1·8) | 0·83 (0·42-1·67, p=0·61) | 7/438 (1·6) | 18/428 (4·2) | 0·40 (0·13-1·22, p=0·11) |
| Iatrogenic preterm birth <34 weeks' gestation | 165/4871 (3·4) | 34/2175 (1·6) | 0·57 (0·17-1·90, p=0·36) | 3/438 (0·7) | 6/428 (1·4) | 0·49 (0·09-2·74, p=0·42) |
| ***Singleton pregnancies*** | | | | | | |
| Preterm birth <34 weeks' gestation | 196/3855 (5·1) | 41/1952 (2·1) | 0·44 (0·18-1·06, p=0·068) | 6/387 (1·6) | 10/366 (2·7) | 0·55 (0·20-1·54, p=0·25) |
| Spontaneous preterm birth <34 weeks' gestation | 65/3701 (1·8) | 22/1932 (1·1) | 0·69 (0·23-2·11, p=0·52) | 5/387 (1·3) | 6/366 (1·6) | 0·75 (0·23-2·51, p=0·65) |
| Iatrogenic preterm birth <34 weeks' gestation | 85/3702 (2·3) | 15/1932 (0·8) | 0·27 (0·06-1·23, p=0·090) | 1/387 (0·3) | 4/366 (1·1) | 0·24 (0·03-2·19, p=0·21) |

Odds ratios were calculated by logistic regression with Huber-White correction for clustering by fetus where multifetal pregnancies were included, with study level as a fixed effect. Adjustments were performed for bile acid concentration at baseline, parity, and number of fetuses. IPD: individual participant data, UDCA: ursodeoxycholic acid, n/N: number affected/total number, aOR: adjusted odds ratio, CI: confidence interval

**Table S8. Perinatal and maternal outcomes according to ursodeoxycholic acid treatment, using individual participant data, excluding single-arm studies (Groups C, D – Appendix p26)**

| | Singleton and multifetal pregnancies | | | Singleton pregnancies | | |
|---|---|---|---|---|---|---|
| **Outcome** | **UDCA n/N (%)** | **No UDCA n/N (%)** | **aOR (95% CI, p value)** | **UDCA n/N (%)** | **No UDCA n/N (%)** | **aOR (95% CI, p value)** |
| Stillbirth | 33/4122 (0·8) | 12/2038 (0·6) | 1·09 (0·51 to 2·33, p=0·82) | 19/2832 (0·7) | 11/1801 (0·6) | 0·71 (0·10 to 4·99, p=0·73) |
| Composite outcome | 2126/4336 (49·0) | 514/2213 (23·2) | 2·23 (1·92 to 2·59, p<0·001) | 996/3013 (33·1) | 353/1955 (18·1) | 0·68 (0·48 to 0·97, p=0·033) |
| Preterm birth <37 weeks' gestation | 2122/4139 (49·1) | 508/2208 (23·0) | 2·26 (1·94 to 2·63, p<0·001) | 992/2997 (33·1) | 347/1952 (17·8) | 0·69 (0·48 to 0·98, p=0·038) |
| Spontaneous preterm birth <37 weeks' gestation | 615/3984 (15·4) | 169/2175 (7·8) | 1·35 (1·06 to 1·71, p=0·015) | 245/2921 (8·4) | 109/1932 (5·6) | 0·54 (0·31 to 0·94, p=0·028) |
| Iatrogenic preterm birth <37 weeks' gestation | 1172/3984 (29·4) | 306/2175 (14·1) | 2·17 (1·82 to 2·59, p<0·001) | 671/2921 (23·0) | 218/1932 (11·3) | 0·88 (0·56 to 1·37, p=0·56) |
| Pre-eclampsia | 203/3153 (6·4) | 121/1574 (7·7) | 1·14 (0·53 to 2·47, p=0·74) | 119/2512 (4·7) | 99/1469 (6·7) | 0·75 (0·26 to 2·21, p=0·61) |
| Unassisted vaginal birth | 1553/3001 (51·7) | 1146/1853 (61·8) | 1·08 (0·83 to 1·40, p=0·59) | 1495/2478 (60·3) | 1121/1740 (64·4) | 1·05 (0·80 to 1·39, p=0·71) |
| Meconium-stained amniotic fluid | 612/3941 (15·5) | 304/1987 (15·3) | 0·98 (0·83 to 1·17, p=0·84) | 419/2964 (15·6) | 266/1760 (15·1) | 0·59 (0·41 to 0·83, p=0·003) |
| Apgar score <7 at 5 minutes | 108/4170 (2·6) | 37/2150 (1·7) | 1·33 (8857 to 2·00, p=0·17) | 69/2878 (2·4) | 32/1902 (1·7) | 0·81 (0·33 to 1·98, p=0·65) |
| Umbilical cord arterial pH <7.0 | 6/1251 (0·5%) | 8/871 (0·9) | 0·51 (0·14 to 1·91, p=0·32) | 3/1024 (0·3%) | 8/779 (1·0) | 0·37 (0·04 to 3·30, p=0·37) |
| Large for gestational age | 399/3443 (11·6) | 220/1432 (15·4) | 0·94 (0·78 to 1·14, p=0·52) | 371/2301 (16·1) | 213/1217 (17·5) | 1·55 (1·07 to 2·26, p=0·021) |
| Small for gestational age | 306/3443 (8·9) | 83/1432 (5·8) | 1·12 (0·83 to 1·52, p=0·47) | 95/2301 (4·1) | 39/1217 (3·2) | 1·33 (0·61 to 2·93, p=0·48) |
| Neonatal unit admission | 1180/4180 (28·2) | 457/2081 (22·0) | 1·58 (1·35 to 1·84, p<0·001) | 645/2887 (22·3) | 371/1842 (20·1) | 0·72 (0·49 to 1·06, p=0·097) |
| Perinatal death | 33/2999 (1·1) | 9/1606 (0·6) | 1·44 (0·63 to 3·27, p=0·39) | 18/2369 (0·8) | 9/1439 (0·6) | 0·54 (0·06 to 4·83, p=0·58) |

Odds ratios were calculated using logistic regression with Huber-White correction, with study level as a fixed effect, and clustering by fetuses for those in multifetal pregnancies. Adjustments were performed for bile acid concentration at baseline, parity, and number of fetuses. For stillbirth, composite outcome (stillbirth or preterm birth), preterm birth, and perinatal outcomes, analyses were performed by number of fetuses; for maternal outcomes, analyses were performed by number of pregnancies. IPD: individual participant data, RCTs: randomised controlled trials, UDCA: ursodeoxycholic acid, n/N: number affected/total number, aOR: adjusted odds ratio, CI: confidence interval

**Table S9. Impact of bile acid concentration at baseline, gestation at diagnosis, and maximum daily ursodeoxycholic acid dose on the composite outcome using individual participant data, from randomised controlled trials (Group E – Appendix p26)**

| | UDCA n/N (%) | No UDCA n/N (%) | aOR (95% CI, p value) | Interaction test |
|---|---|---|---|---|
| **Bile acids (µmol/L)** | **Composite outcome** | | | |
| <40 | 43/323 (13·3) | 64/319 (20·1) | 0·63 (0·37-1·06, p=0·079) | p=0·74 |
| ≥40 | 32/116 (27·6) | 43/110 (39·1) | 0·56 (0·29-1·09, p=0·086) | |
| <100 | 66/410 (16·1) | 98/403 (24·3) | 0·58 (0·38-0·90, p=0·014) | p=0·79 |
| ≥100 | 9/29 (31·0) | 9/26 (34·6) | 0·75 (0·20-2·81, p=0·67) | |
| **Diagnosis gestation (weeks)** | | | | |
| <32 | 35/117 (29·9) | 38/115 (33·0) | 0·76 (0·39-1·48, p=0·42) | p=0·38 |
| ≥32 | 26/263 (9·9) | 43/250 (17·2) | 0·51 (0·28-0·95, p=0·034) | |
| **UDCA daily dosage (g/day)** | | **Placebo** | | |
| <1 | 7/56 (12·5) | 14/46 (30·4) | 0·18 (0·05-0·70, p= 0·013) | p=0·069 |
| ≥1 | 68/383 (17·8) | 93/383 (24·3) | 0·69 (0·45-1·06, p=0·094) | |

Analyses performed using individual participant data from participants of randomised controlled trials. Composite outcome: any one of stillbirth or preterm birth before 37/40, recorded once for each fetus. Odds ratios were calculated using logistic regression with Huber-White correction for clustering by fetus, adjustments were performed for parity and number of fetuses. UDCA: ursodeoxycholic acid, aOR: adjusted odds ratio

**Table S10. Impact of bile acid concentration at baseline on perinatal outcomes using individual participant data, from all studies (Group A – Appendix p26)**

| Outcome | Baseline bile acid concentration (μmol/L) n/N (%) | | | Comparison <40 vs ≥40 aOR (95% CI, p value) | Comparison <100 vs ≥100 aOR (95% CI, p value) |
|---|---|---|---|---|---|
| | <40 | 40-99 | ≥100 | | |
| Neonatal unit admission | 521/2478 (21·0) | 312/1266 (24·6) | 114/413 (27·6) | 1·43 (1·15-1·78, p=0·001) | 1·64 (1·23-2·19, p=0·001) |
| Meconium-stained amniotic fluid | 310/2517 (12·3) | 223/1278 (17·4) | 109/406 (26·8) | 1·93 (1·54-2·42, p<0·001) | 2·27 (1·69-3·04, p<0·001) |
| Umbilical cord arterial pH<7·0 | 4/467 (0·9) | 3/398 (0·8) | 0/163 (0·0) | 1·70 (0·24-12·14, p=0·60) | 0·53 (0-3·54, p=0·57) |
| Apgar score <7 at 5 minutes | 79/2581 (3·1) | 50/1310 (3·8) | 16/396 (4·0) | 1·23 (0·81-1·87, p=0·33) | 1·60 (0·91-2·83, p=0·11) |
| Neonatal death | 1/1830 (0·1) | 3/1117 (0·3) | 4/371 (1·1) | 10·80 (0·38-306·53, p=0·16) | 8·31 (2·13-32·41, p=0·002) |
| Perinatal death | 8/1830 (0·4) | 9/1117 (0·8) | 13/371 (3·5) | 2·78 (0·99-7·82, p=0·053) | 5·94 (2·65-13·29, p<0·001) |
| Small for gestational age | | | | | |
| Singleton | 50/1470 (3·4) | 35/895 (3·9) | 12/332 (3·6) | 1·24 (0·75-2·04, p=0·40) | 0·90 (0·44-1·81, p=0·76) |
| Multifetal | 159/824 (19·3) | 64/351 (18·2) | 11/60 (18·3) | 0·89 (0·59-1·34, p=0·59) | 0·79 (0·37-1·66, p=0·53) |
| Large for gestational age | | | | | |
| Singleton | 233/1470 (15·9) | 158/895 (17·7) | 36/332 (10·8) | 0·87 (0·66-1·13, p=0·30) | 0·53 (0·36-0·79, p=0·001) |
| Multifetal | 17/824 (2·1) | 7/351 (2·0) | 2/60 (3·3) | 1·39 (0·50-3·88, p=0·53) | 3·55 (0·24-53·23, p=0·36) |

Analyses were performed using individual participant data from all studies. Logistic regression was performed with Huber-White correction for clustering, as the multilevel model failed to converge. n/N: number with outcome/total number, aOR: adjusted odds ratio, CI: confidence interval.

**Table S11. Maternal outcomes according to ursodeoxycholic acid treatment using individual participant data, from all studies (Groups A, E – Appendix p26)**

| Outcome | | All studies IPD | | | Randomised controlled trials IPD | | |
|---|---|---|---|---|---|---|---|
| | | **UDCA n/N (%)** | **No UDCA n/N (%)** | **aOR (95% CI, p value)** | **UDCA n/N (%)** | **No UDCA n/N (%)** | **aOR (95% CI, p value)** |
| Birth onset induced | | 1358/2358 (57·6) | 785/1100 (71·4) | 1·11 (0·85-1·46, p=0·44) | 274/412 (66·5) | 253/397 (63·7) | 1·12 (0·83-1·51, p=0·48) |
| Postpartum haemorrhage | | 714/2456 (29·1) | 363/1069 (34·0) | 1·06 (0·81-1·39, p=0·67) | 135/378 (35·7) | 137/370 (37·0) | 0·97 (0·71-1·33, p=0·85) |
| Mode of delivery | | | | | | | |
| | Unassisted vaginal birth | 1926/3842 (50·1) | 1146/1853 (61·8) | Referent | 261/412 (63·3) | 253/397 (63·7) | Referent |
| | Assisted vaginal delivery | 196/3842 (5·1) | 123/1853 (6·6) | 0·85 (0·64-1·12, p=0·24) | 29/412 (7·0) | 40/397 (10·1) | 0·68 (0·40-1·14, p=0·14) |
| | Elective caesarean section | 805/3842 (21·0) | 240/1853 (13·0) | 1·18 (0·97-1·44, p=0·092) | 77/412 (18·7) | 63/397 (15·9) | 1·27 (0·87-1·87, p=0·22) |
| | Emergency caesarean section | 449/3842 (11·7) | 158/1853 (8·5) | 0·84 (0·65-1·67, p=0·15) | 37/412 (9·0) | 37/397 (9·3) | 0·97 (0·58-1·61, p=0·90) |
| | Unknown caesarean section | 466/3842 (12·1) | 186/1853 (10·0) | 1·29 (1·02-1·64, p=0·036) | 8/412 (1·9) | 4/397 (1·0) | 2·01 (0·59-6·81, p=0·27) |

Adjustments were made for parity, multifetal pregnancy, baseline bile acid concentration, using multilevel modelling to compare with study and mother as separate levels. Women who had a stillbirth were not included in onset of birth or mode of delivery analyses. IPD: individual participant data, UDCA: ursodeoxycholic acid, n/N: number with outcome/total number, aOR: adjusted odds ratio, CI: confidence interval, n/a: not applicable (insufficient numbers reported for analysis).

**Table S12. Impact of bile acid concentration at baseline on maternal pregnancy outcomes using individual participant data, from all studies (Group A – Appendix p26)**

| Outcome | Baseline bile acid concentration (μmol/L) n/N (%) | | | Comparison <40 vs ≥40 aOR (95% CI, p value) | Comparison <100 vs ≥100 aOR (95% CI, p value) |
|---|---|---|---|---|---|
| | <40 | 40-99 | ≥100 | | |
| Birth onset induced | 925/1678 (55·1) | 342/598 (57·2) | 113/185 (61·1) | 0·81 (0·66-1·01, p=0·059) | 0·61 (0·43-0·88, p=0·007)* |
| Unassisted vaginal birth | 1038/2117 (49·0) | 393/807 (48·7) | 131/234 (56·0) | 1·07 (0·90-1·28, p=0·45) | 1·11 (0·82-1·50, p=0·51) |
| Caesarean section | 958/2117 (45·3) | 358/807 (44·4) | 85/234 (36·3) | 0·93 (0·78-1·12, p=0·45) | 0·97 (0·71-1·33, p=0·86) |
| Pre-eclampsia | 94/1661 (5·7) | 52/1008 (5·2) | 16/324 (4·9) | 0·69 (0·45-1·05, p=0·082) | 0·83 (0·47-1·48, p=0·53) |
| Postpartum haemorrhage | 536/1512 (35·4) | 187/549 (34·1) | 54/170 (31·8) | 0·81 (0·65-1·01, p=0·057) | 0·86 (0·59-1·25, p=0·42) |

Analyses performed using individual participant data from all studies. Odds ratios are calculated using multilevel models, with mother and study considered as separate levels, including women from all studies. Adjustment is made for parity and number of fetuses. Women who had a stillbirth are not included in onset of birth or mode of delivery analyses. *Adjustment reversed the direction of the association, this was largely due to study effect. n/N: number with outcome/total number, aOR: adjusted odds ratio, CI: confidence interval

**Table S13. Randomised controlled trials included in the aggregate data meta-analysis (Group G – Appendix p26)**

| Name of study | UDCA group | UDCA number | Comparator group | Comparator number | Use of masking | Intervention duration (days) | Included in IPD meta-analysis |
|---|---|---|---|---|---|---|---|
| Ai et al., 2002[88] | UDCA | 15 | Dexamethasone / Glucose + vitamin C + inosine + phenobarbital | 31 | No | 14 | No |
| Binder et al., 2006[41] | UDCA / UDCA + SAMe | 53 | SAMe | 25 | No | Until birth | No |
| Chappell et al., 2012[12] | UDCA | 56 | Placebo | 55 | Yes | Until birth | Yes |
| Chappell et al., 2019[13] | UDCA | 305 | Placebo | 300 | Yes | Until birth | Yes |
| Diaferia et al., 1996[89] | UDCA | 8 | Placebo | 8 | Yes | 20 | No |
| Floreani et al., 1996[90] | UDCA | 10 | SAMe | 10 | No | Until birth | No |
| Glantz et al., 2005[53] | UDCA | 47 | Placebo / Dexamethasone | 83 | Yes | 21 | No |
| Joutsiniemi et al., 2014[91] | UDCA | 10 | Placebo | 8 | Yes | 14 | No |
| Kondrackiene et al., 2005[25] | UDCA | 42 | Cholestyramine | 42 | No | 14 | Yes |
| Liu et al., 2006[65] | UDCA | 34 | Glucose + vitamin C + inosine | 34 | No | 14 | No |
| Nicastri et al., 1998[69] | UDCA / UDCA + SAMe | 16 | SAMe / Placebo | 16 | No | 20 | No |
| Palma et al., 1997[92] | UDCA | 8 | Placebo | 7 | Yes | 21 | No |
| Roncaglia et al., 2004[32] | UDCA | 24 | SAMe | 22 | No | Until birth | Yes |
| Zhang et al., 2015[86] | UDCA / UDCA + SAMe | 82 | SAMe | 38 | No | Until birth | No |

UDCA: ursodeoxycholic acid, IPD: individual participant data, SAMe: S-Adenosylmethionine

**Figure S1. Schematic of dataset subgroups**

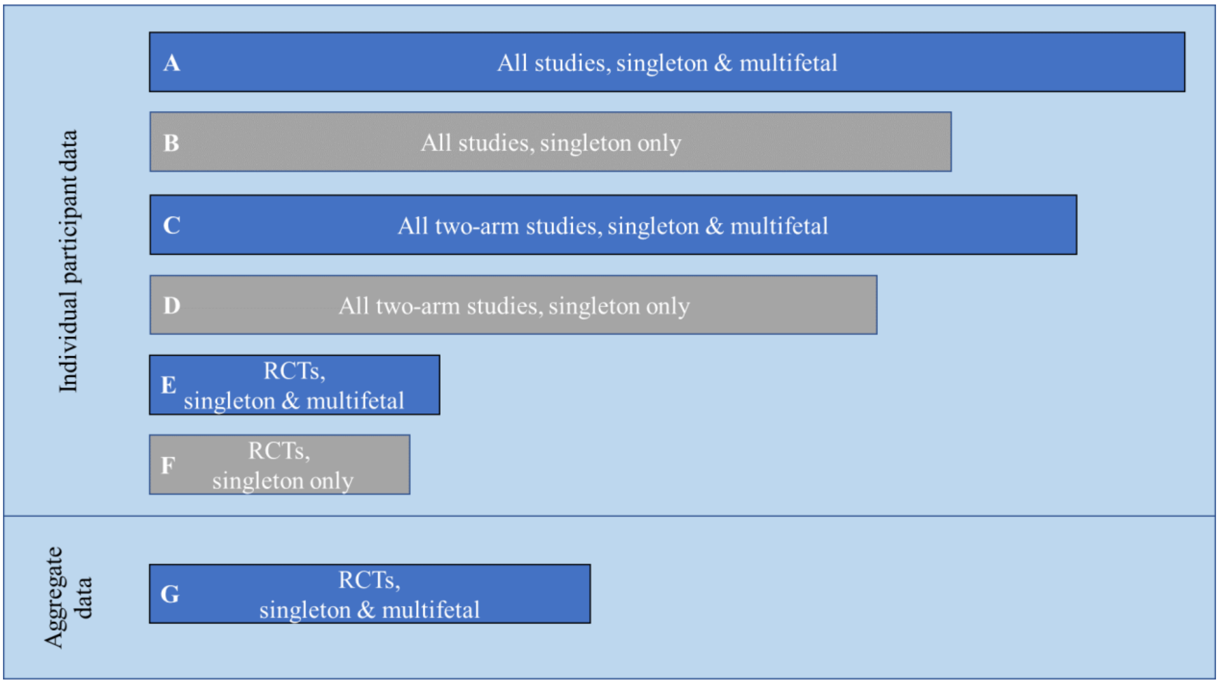


RCTs: randomised controlled trial. Boxes not drawn to scale.

**Figure S2. Rates of stillbirth and preterm birth for women receiving or not receiving ursodeoxycholic acid during their pregnancies, using individual participant data from all two-arm studies (Group C – Appendix p26)**

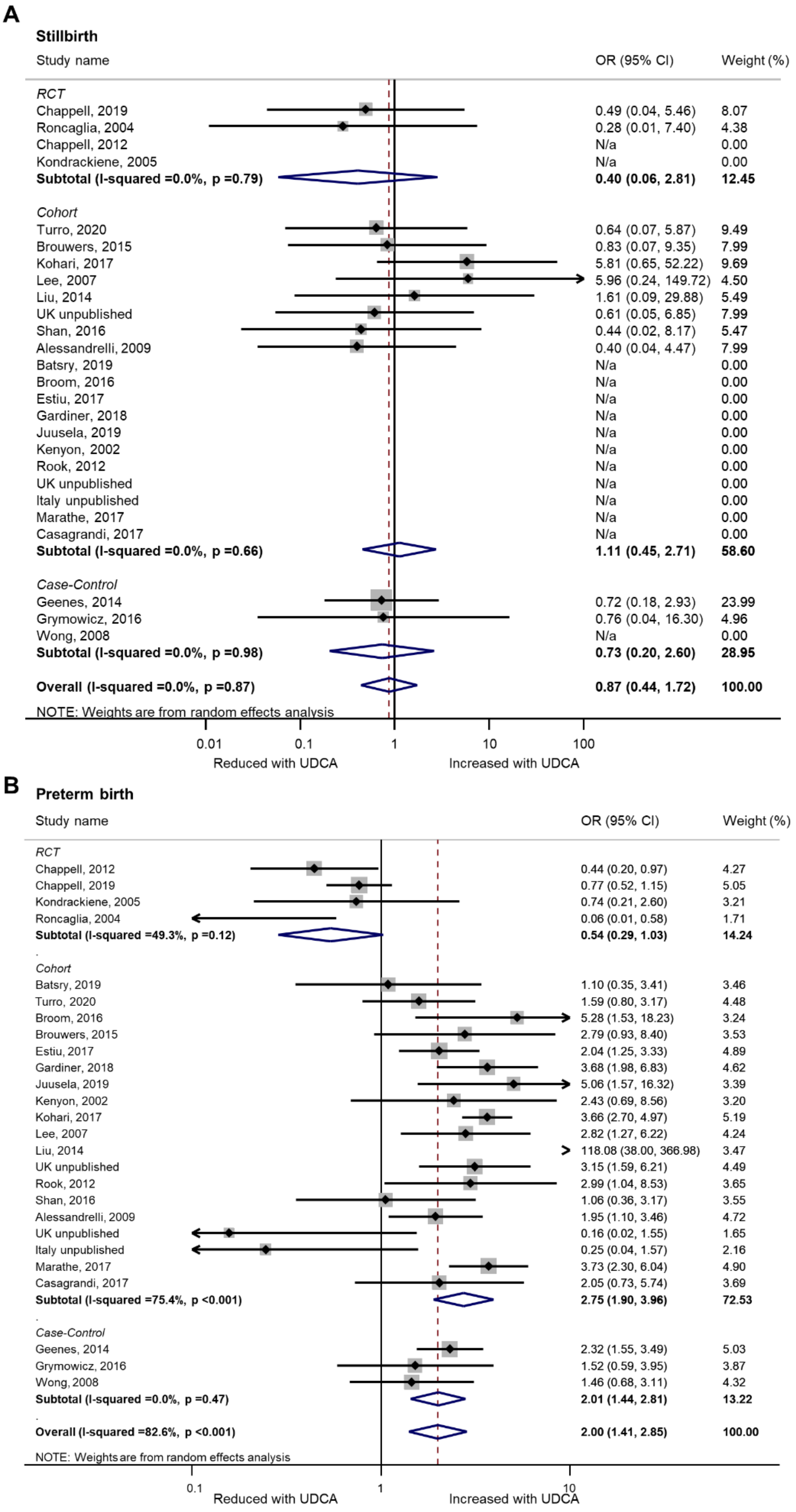

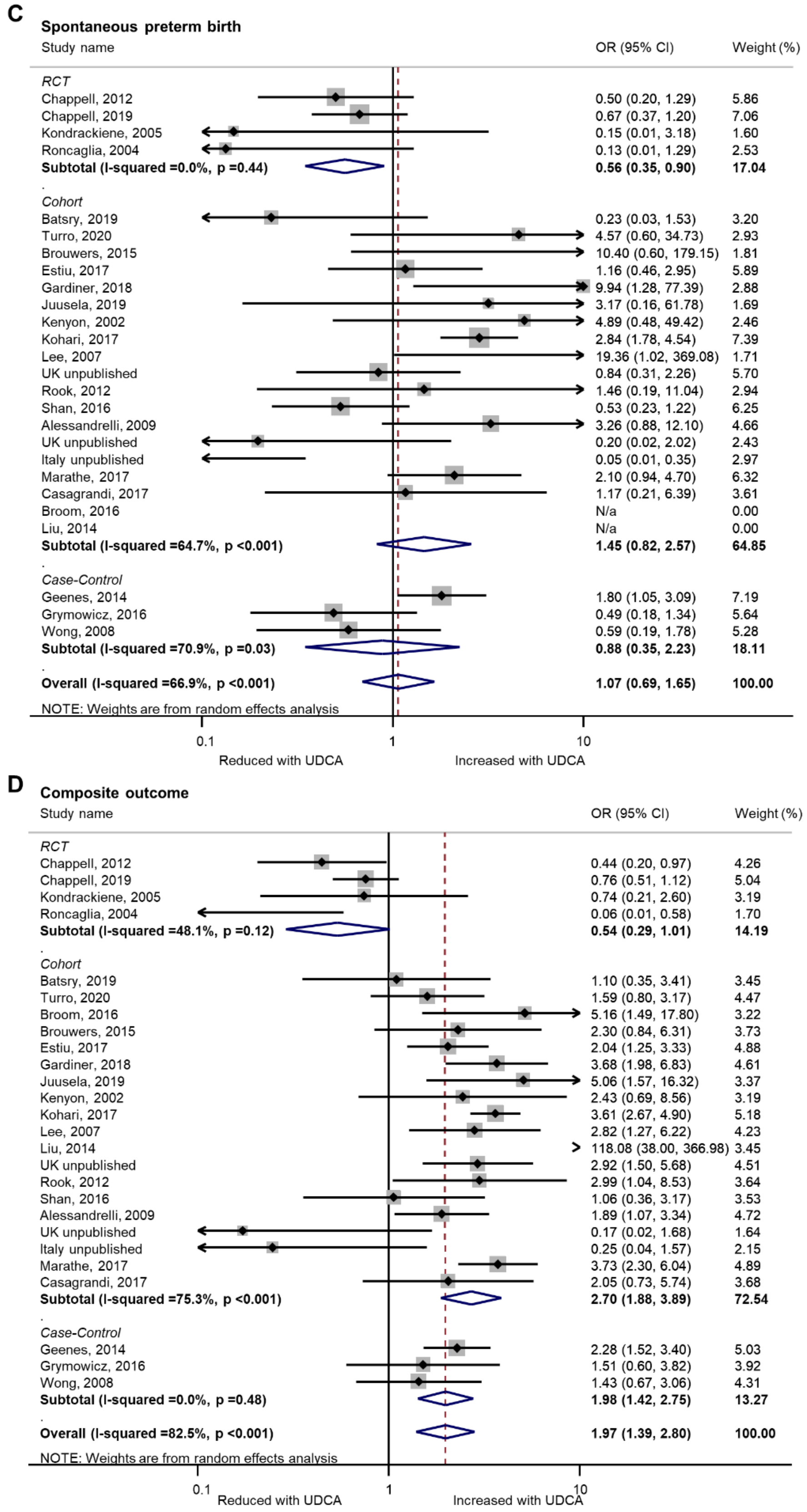


Comparisons performed by study type using a two-stage meta-analysis, with OR calculated for each study separately and combined with a random effects model. Only studies with participants in both arms (UDCA / no UDCA) were included. A: Stillbirth, B: All preterm birth <37 gestational weeks, C: Spontaneous preterm birth <37 gestational weeks, D: Composite outcome: any one of stillbirth or preterm birth. OR: odds ratio, CI: confidence interval, RCT: randomised controlled trial, UDCA: ursodeoxycholic acid, N/a: not applicable

**Figure S3. Proportion and incidence of stillbirths and total number of singleton pregnancies by peak total bile acid concentration and ursodeoxycholic acid treatment using individual participant data, from all studies (Group B – Appendix p26)**

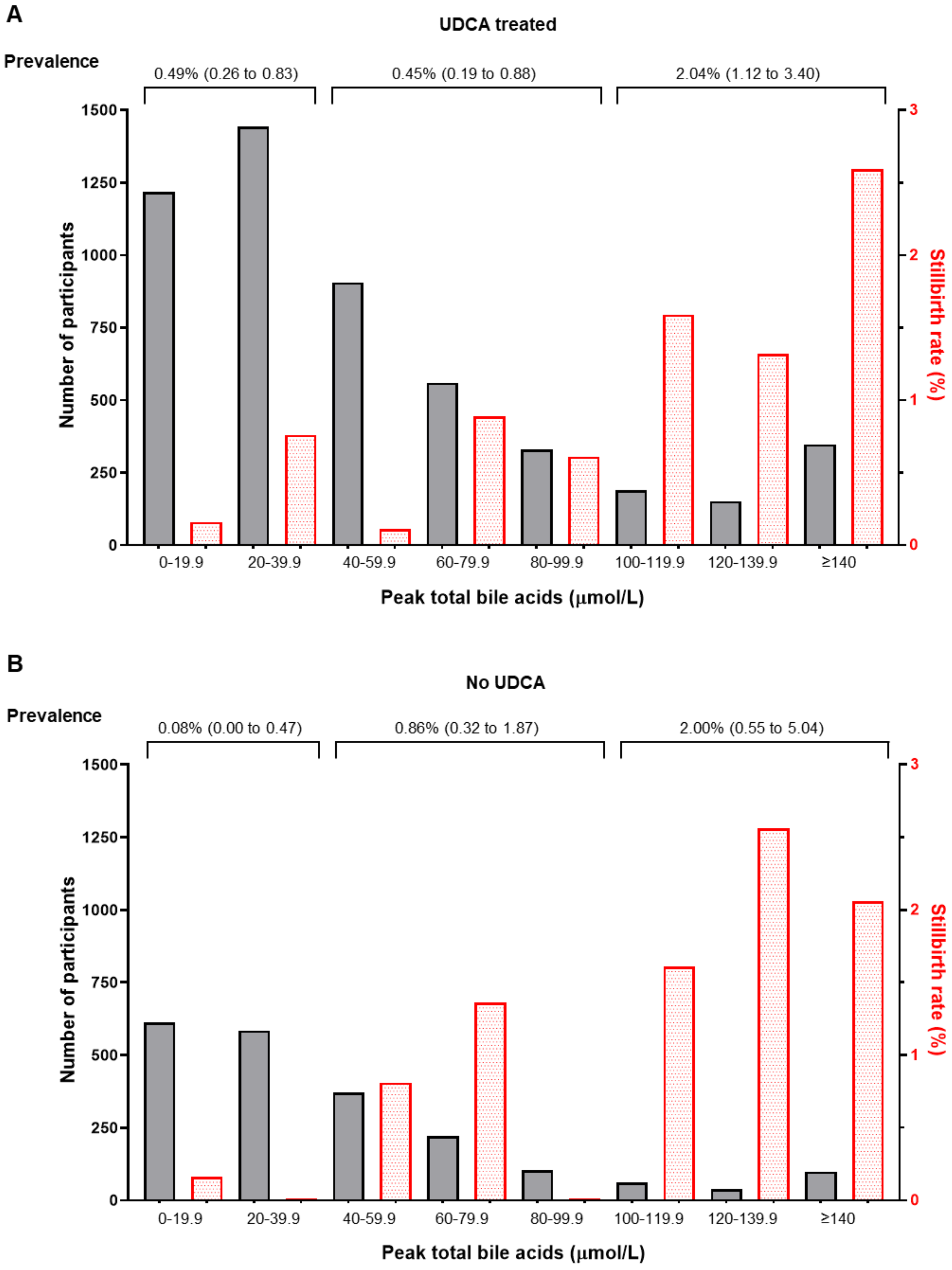


A: women treated with UDCA, B: women not treated with UDCA. Number of women with ICP (grey bars) and stillbirth proportion (red bars) by peak total bile acid category. Stillbirth prevalence from participants in all studies using individual participant data, by total bile acid groups <40 µmol/L, 40-99·9 µmol/L, and ≥100 µmol/L (95% confidence interval). UDCA: ursodeoxycholic acid

**Figure S4. Receiver operating characteristic curves to determine the association between stillbirth and peak bile acid concentration for women with singleton pregnancies, using individual participant data, from all studies (Group B – Appendix p26)**

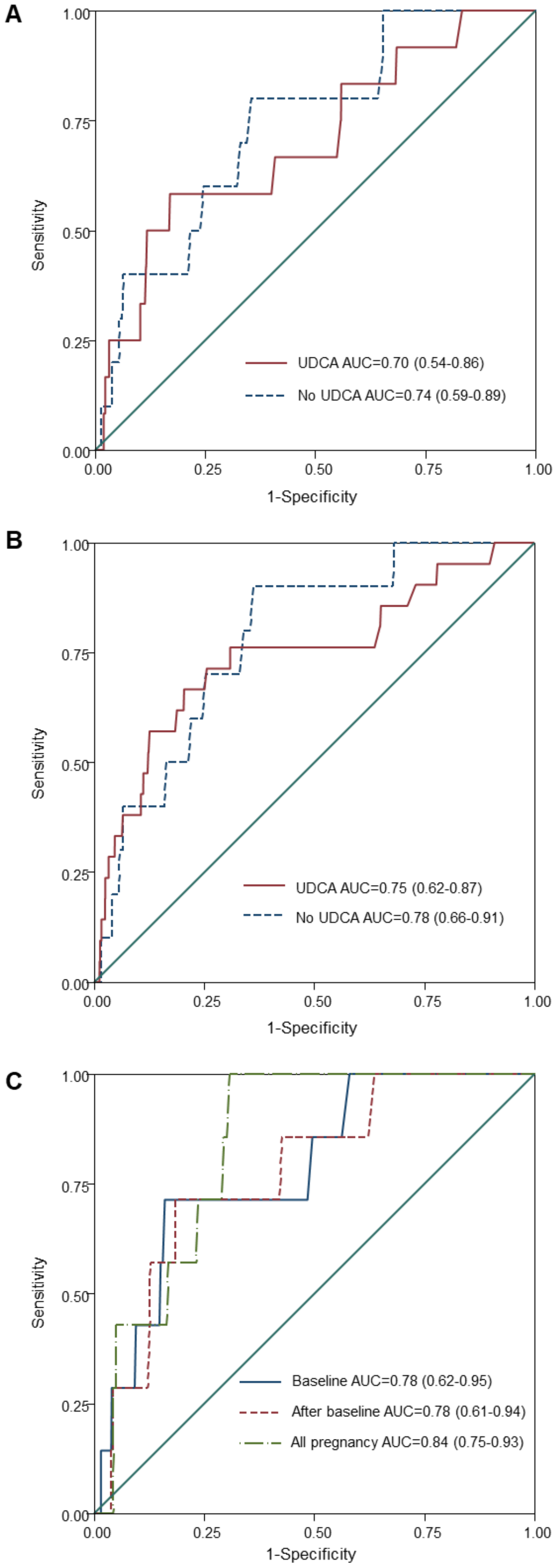


A: Effect of UDCA on association between stillbirth and peak bile acids post baseline. No difference was found between women receiving UDCA or no UDCA (p=0·72). UDCA n=2138, no UDCA n=1821

B: Effect of UDCA on association between stillbirth and peak bile acids for all of the pregnancy. No difference was found between women receiving UDCA or no UDCA (p=0·69). UDCA n=3775, no UDCA n=1936

C: Effect of timing of peak bile acid measurement on association with stillbirth. No difference was found between timings and association of stillbirth with bile acid levels (p=0·15). N=1417

Baseline: highest bile acid measurement taken before starting intervention, placebo or observation. After baseline: highest bile acid measurement taken after starting intervention, placebo or observation. All pregnancy: highest bile acid measurement recorded throughout the pregnancy. AUC: area under the curve (95% confidence interval), UDCA: ursodeoxycholic acid

**Figure S5. Kaplan-Meier survival plots of spontaneous preterm birth rate by gestational week of birth, according to ursodeoxycholic acid use and disease severity at randomisation for women with singleton pregnancies, using individual participant data from randomised controlled trials (Group F – Appendix p26)**

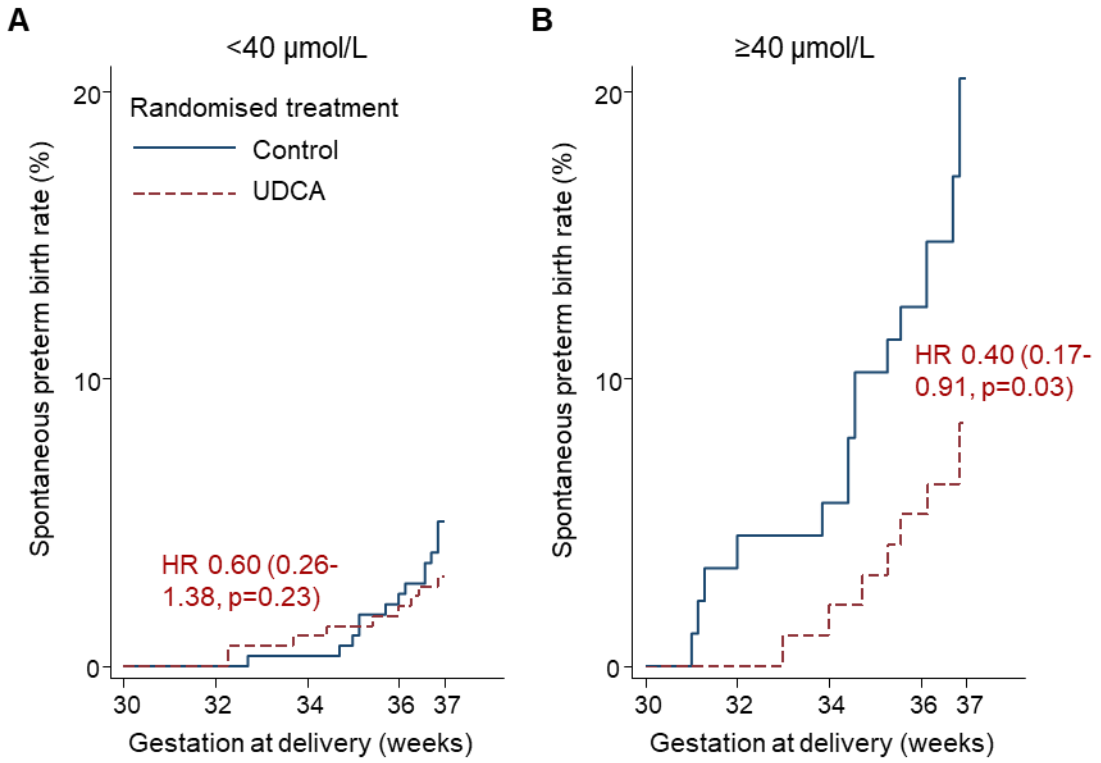


A, B: Women with baseline bile acid concentration <40 and ≥40 µmol/L respectively, hazard ratios comparing the women randomised to UDCA with those randomised to placebo, calculated accounting for study effect. Interaction test for the groups: p=0·61

Women were censored at 37/40 if still pregnant at that time, or where birth onset was not spontaneous. HR: hazard ratio, UDCA: ursodeoxycholic acid. HR presented with 95% confidence interval

**Figure S6. Kaplan-Meier survival plots of iatrogenic preterm birth rate by gestational week of birth, according to ursodeoxycholic acid use and disease severity at randomisation for women with singleton pregnancies, using individual participant data, from randomised controlled trials (Group F – Appendix p26)**

**A**

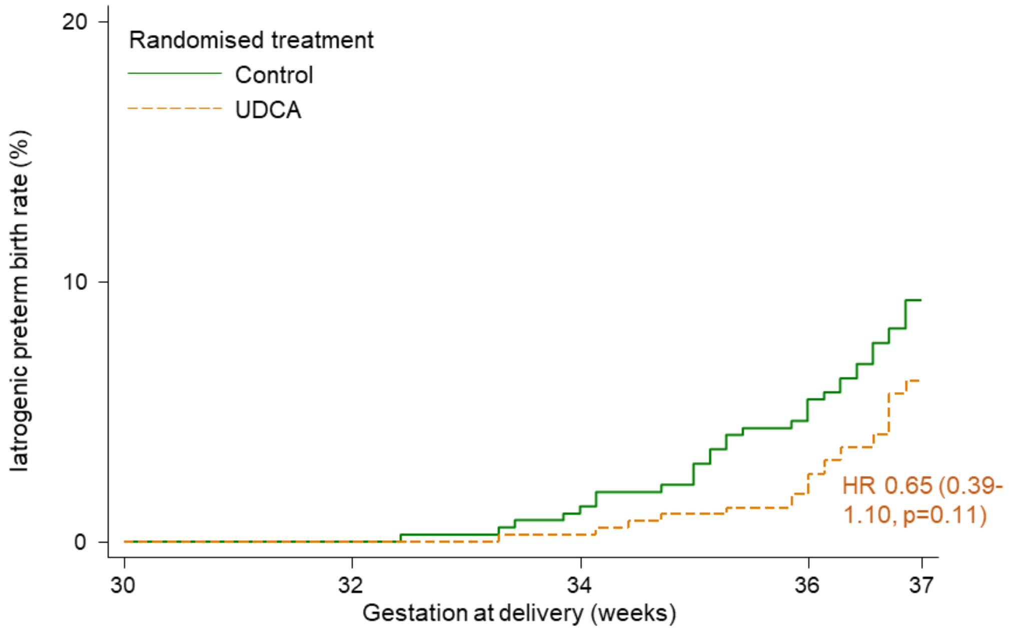


**B**

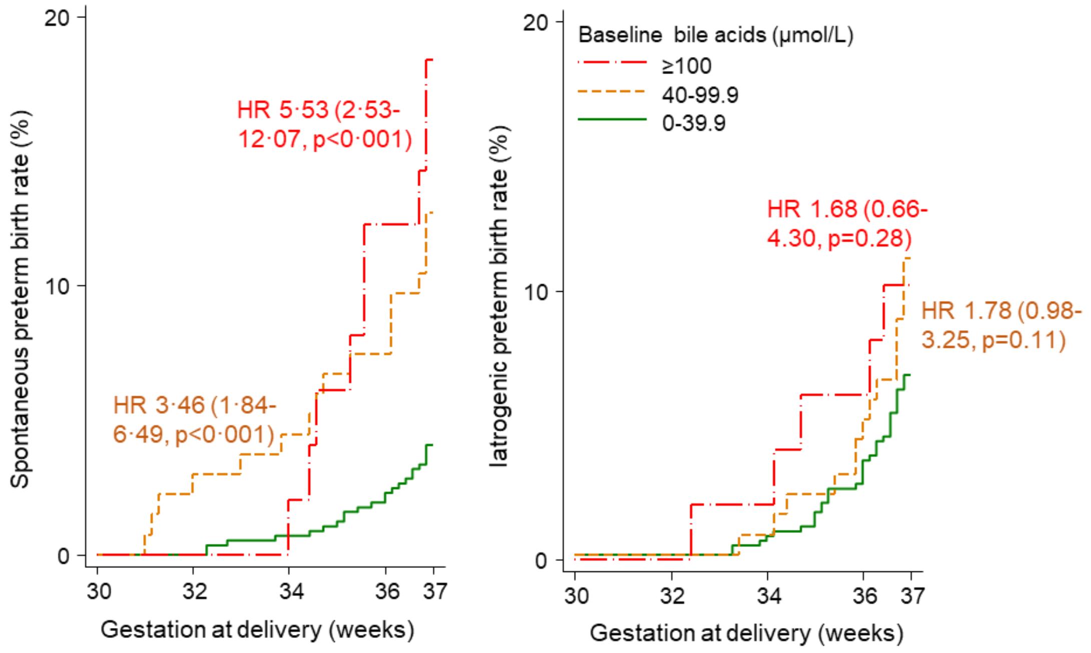


A: All women

B: All women by baseline bile acid concentration, hazard ratios comparing women with baseline bile acids 40-99.9 (red) and ≥100 µmol/L (orange) to those with baseline <40 µmol/L. The graph of spontaneous preterm birth is reproduced from Figure 2 for comparison.

Women were censored at 37/40 if still pregnant at that time, or where birth onset was not clinician-induced. Hazard ratios were calculated accounting for study effect and presented with 95% confidence interval. HR: hazard ratio, UDCA: ursodeoxycholic acid.

**Figure S7. Funnel plots of studies in the aggregate data meta-analysis of randomised controlled trials (Group G – Appendix p26)**

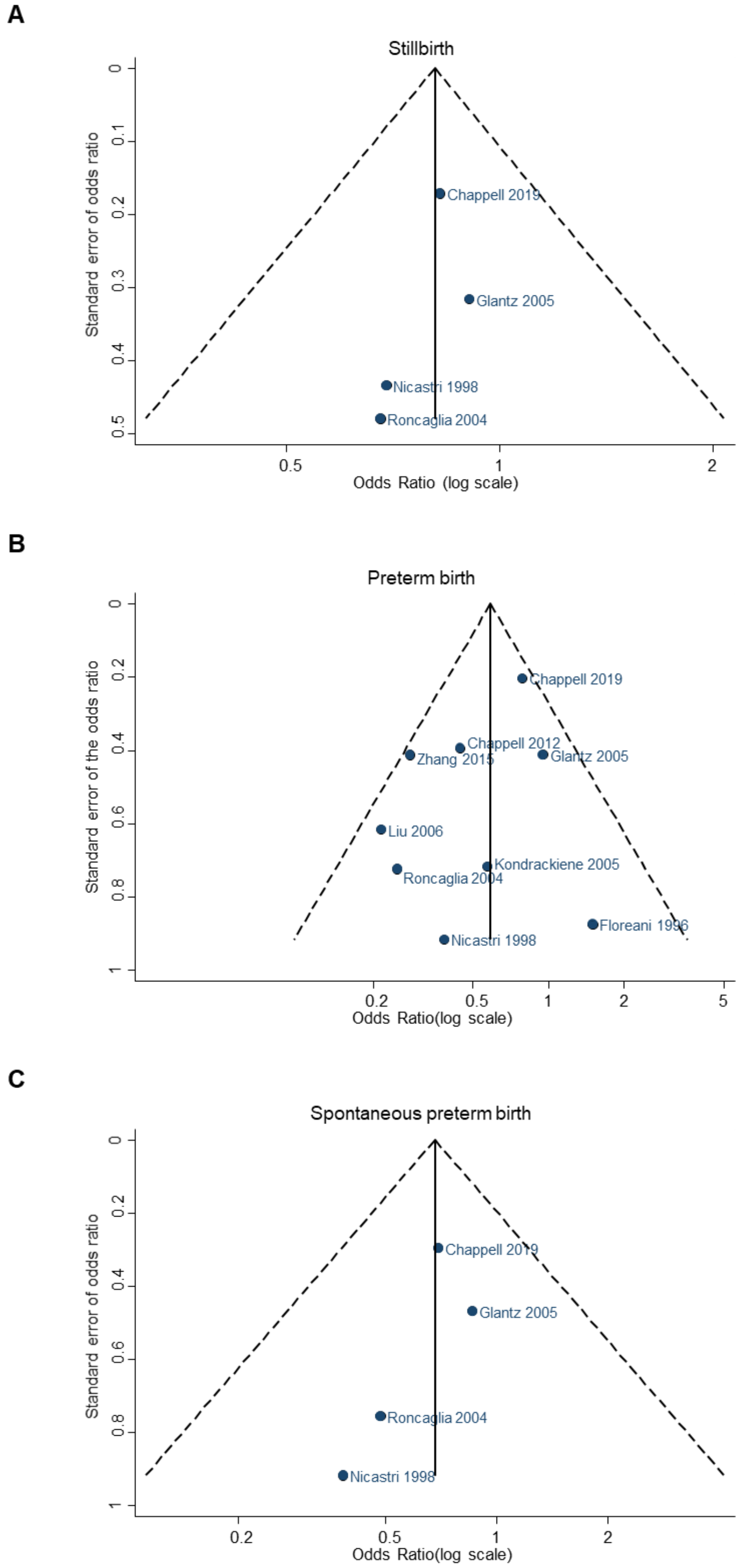


Funnel plots are presented showing pseudo 95% confidence intervals. A: stillbirth, B: preterm birth, C: spontaneous preterm birth

**Supplementary References**


1 Rabe-Hesketh S, Skrondal A. Multilevel and Longitudinal Modelling Using Stata. 3rd ed. 2012; College Station, TX: Stata Press.

2 National, Heart, Lung, and Blood Institute. NHLBI Quality Assessment Tool for Observational Cohort and Cross-Sectional Studies. https://www.nhlbi.nih.gov/health-topics/study-quality-assessment-tools downloaded on 26 Feb 2020.

3 Ovadia C, Seed PT, Sklavounos A, et al. Association of adverse perinatal outcomes of intrahepatic cholestasis of pregnancy with biochemical markers: results of aggregate and individual patient data meta-analyses. *Lancet* 2019; **393**:899–909.

4 Watson HA, Carter J, Seed PT, Tribe RM, Shennan AH. The QUiPP App: a safe alternative to a treat-all strategy for threatened preterm labor. *Ultrasound Obstet Gynecol* 2017; **50:** 342–346.

5 Alessandrelli F, Azzaroli F, Feletti V, et al. A retrospective study on intrahepatic cholestasis of pregnancy: markers of premature delivery. *Dig Liver Dis* 2009; **41:** S106–S107.

6 Bacq Y, le Besco M, Lecuyer AI, et al. Ursodeoxycholic acid therapy in intrahepatic cholestasis of pregnancy: Results in real-world conditions and factors predictive of response to treatment. *Dig Liver Dis* 2017; **49:** 63–69.

7 Batsry L, Zloto K, Kalter A, Baum M, Mazaki-Tovi S, Yinon Y. Perinatal outcomes of intrahepatic cholestasis of pregnancy in twin versus singleton pregnancies: is plurality associated with adverse outcomes? *Arch Gynecol Obstet* 2019; **300:** 881–887.

8 Broom K, Kane S. The Incidence, Management and Outcomes of Obstetric Cholestasis at the Royal Women's Hospital Victoria – A Retrospective Study. *ANZJOG* 2016; **56(suppl1):** 10.

9 Brouwers L, Koster M, Page-Christiaens G, et al. Intrahepatic cholestasis of pregnancy: maternal and fetal outcomes associated with elevated bile acid levels. *Am J Obstet Gynecol* 2015; **212:** 100.e1–100.e7.

10 Brun-Furrer R, Winter K, Schäffer L, Zimmermann R, Burkhardt T, Haslinger C. Postpartum Blood Loss in Women Treated for Intrahepatic Cholestasis of Pregnancy. *Obstet Gynecol* 2016; **128:** 1048–1052.

11 Casagrandi D, Maksym K, Tetteh A, Sarquis R, Attilakos G. Diagnostic pathway, management and outcomes of women with obstetric cholestasis at UCLH. *Br J Obstet Gynaecol* 2017; **124(S2):** P.MM.85.

12 Castaño G, Lucangioli S, Sookoian S, et al. Bile acid profiles by capillary electrophoresis in intrahepatic cholestasis of pregnancy. *Clin Sci* 2006; **110:** 459–465.

13 Chappell LC, Gurung V, Seed P, Chambers J, Williamson C, Thornton J. Ursodeoxycholic acid versus placebo, and early term delivery versus expectant management, in women with intrahepatic cholestasis of pregnancy: semifactorial randomised clinical trial. *BMJ* 2012; **344:** e3799.

14 Chappell LC, Bell JL, Smith A, et al. Ursodeoxycholic acid versus placebo in women with intrahepatic cholestasis of pregnancy (PITCHES): a randomised controlled trial. *Lancet* 2019; **394:** 849–860.

15 Cui Y, Xu B, Zhang X, He Y, Shao Y, Ding M. Diagnostic and therapeutic profiles of serum bile acids in women with intrahepatic cholestasis of pregnancy-a pseudo-targeted metabolomics study. *Clin Chim Acta* 2018; **483:** 135–141.

16 Estiú MC, Frailuna MA, Otero C, et al. Relationship between early onset severe intrahepatic cholestasis of pregnancy and higher risk of meconium-stained fluid. *PLoS One* 2017; **12:** e0176504.

17 Gardiner FW, Mccuaig R, Arthur C, et al. The prevalence and pregnancy outcomes of intrahepatic cholestasis of pregnancy: A retrospective clinical audit review. *Obstet Med* 2019; **12:** 123–128.

18 Geenes V, Chappell LC, Seed PT, Steer PJ, Knight M, Williamson C. Association of severe intrahepatic cholestasis of pregnancy with adverse pregnancy outcomes: A prospective population-based case-control study. *Hepatology* 2014; **59:** 1482–1491.

19 Grymowicz M, Czajkowski K, Smolarczyk R. Pregnancy course in patients with intrahepatic cholestasis of pregnancy treated with very low doses of ursodeoxycholic acid. *Scand J Gastroenterol* 2016; **51:** 78–85.

20 Günaydin B, Bayram M, Altuğ M, Cevher S, Bozkurt N. Retrospective analysis of maternal, fetal, and neonatal outcomes of intrahepatic cholestasis of pregnancy at Gazi University. *Turkish J Med Sci* 2017; **47:** 583–586.

21 Juusela AL, Cordero L, Gimovsky ML, Nazir M. Correlation of bile acids and aspartate-aminoternsferase with outcomes in cholestasis of pregnancy. *J Neonatal Perinatal Med* 2019, doi: 10.3233/NPM-190276.

22 Kebapcilar AG, Taner CE, Kebapcilar L, Bozkaya G. High mean platelet volume, low-grade systemic coagulation, and fibrinolytic activation are associated with pre-term delivery and low APGAR score in intrahepatic cholestasis of pregnancy. *J Matern Neonatal Med* 2010; **23:** 1205–1210.

23 Kenyon A, Piercy C, Girling C, Williamson C, Tribe RM, Shennan A. Pruritus may precede abnormal liver function tests in pregnant women with obstetric cholestasis: A longitudinal analysis. *Br J Obstet Gynaecol* 2001; **108:** 1190–1192.
24 Kohari KS, Carroll R, Capogna S, Ditchik A, Fox NS, Ferrara LA. Outcome after implementation of a modern management strategy for intrahepatic cholestasis of pregnancy. *J Matern Neonatal Med* 2017; **30:** 1342–1346.
25 Kondrackiene J, Beuers U, Kupcinskas L. Efficacy and Safety of Ursodeoxycholic Acid Versus Cholestyramine in Intrahepatic Cholestasis of Pregnancy. *Gastroenterology* 2005; **129:** 894–901.
26 Lee R, Kwok K, Ingles S, et al. Association of bile acid concentrations with adverse pregnancy outcome in an era of aggressive management for intrahepatic cholestasis of pregnancy. *Am J Obstet Gynecol* 2007; **197:** S93.
27 Liu X, Landon MB, Chen Y, Cheng W. Perinatal outcomes with intrahepatic cholestasis of pregnancy in twin pregnancies. *J Matern Neonatal Med* 2016; **29:** 2176–2181.
28 Madazli R, Yuksel MA, Oncul M, Tuten A, Guralp O, Aydin B. Pregnancy outcomes and prognostic factors in patients with intrahepatic cholestasis of pregnancy. *J Obstet Gynaecol* 2015; **35:** 358–361.
29 Majewska A, Godek B, Bomba-Opon D, Wielgos M. Association between intrahepatic cholestasis in pregnancy and gestational diabetes mellitus. A retrospective analysis. *Ginekol Pol* 2019; **90:** 458–463.
30 Marathe JA, Lim WH, Metz MP, Scheil W, Dekker GA, Hague WM. A retrospective cohort review of intrahepatic cholestasis of pregnancy in a South Australian population. *Eur J Obstet Gynecol Reprod Biol* 2017; **218:** 33–38.
31 Öztekin D, Aydal I, Oztekin O, Okcu S, Borekci R, Tinar S. Predicting Fetal Asphyxia in Intrahepatic Cholestasis of Pregnancy. *Arch Gynecol Obstet* 2009; **280:** 975–979.
32 Roncaglia N, Locatelli A, Arreghini A, et al. A randomised controlled trial of ursodeoxycholic acid and S-adenosyl-l-methionine in the treatment of gestational cholestasis. *Br J Obstet Gynaecol* 2004; **111:** 17–21.
33 Rook M, Vargas J, Caughey A, Bacchetti P, Rosenthal P, Bull L. Fetal Outcomes in Pregnancies Complicated by Intrahepatic Cholestasis of Pregnancy in a Northern California Cohort. *PLoS One* 2012; **7:** 3–8.
34 Shan D, Hu Y, Qiu P, et al. Intrahepatic Cholestasis of Pregnancy in Women With Twin Pregnancy. *Twin Res Hum Genet* 2016; **19:** 697–707.
35 Turro E, Astle WJ, Megy K et al. Whole-genome sequencing of patients with rare diseases in a national health system. *Nature* 2020; **583:** 96-102.
36 Wong LFA, Shallow H, O'Connell MP. Comparative study on the outcome of obstetric cholestasis. *J Matern Neonatal Med* 2008; **21:** 327–330.
37 Aksan Desteli G, Sahin-Uysal N, Cok T, Gulumser C, Kalayci H, Yanik FF. First trimester maternal serum PAPP-A levels and associated pregnancy complications in intrahepatic cholestasis of pregnancy. *Clin Exp Obstet Gynecol* 2016; **43:** 673–677.
38 Al-Obaidly S, Salama H, Olayemi Olukade T, Al-Qubaisi M, Al Rifai H. Obstetrics and neonatal outcomes of intrahepatic cholestasis of pregnancy: A population-based study. *Eur J Obs Gynecol Repro Biol* 2019; **234:** E155-156.
39 Al Shobaili HA, Hamed HO, Al Robaee A, Alzolibani AA, Amin AF, Ahmad SR. Obstetrical and fetal outcomes of a new management strategy in patients with intra-hepatic cholestasis of pregnancy. *Arch Gynecol Obstet* 2011; **283:** 1219–1225.
40 Alsulyman OM**,** Ouzounian JG, Ames-Castro M, Goodwin TM. Intrahepatic cholestasis of pregnancy: perinatal outcome associated with expectant management. *Am J Obstet Gynecol* 1996**; 175**: 957–960.
41 Binder T, Salaj P, Zima T, Vítek L. Randomized prospective comparative study of ursodeoxycholic acid and S-adenosyl-L-methionine in the treatment of intrahepatic cholestasis of pregnancy. *J Perinat Med* 2006; **34:** 383–391.
42 Bolukbas FF, Bolukbas C, Y Balaban H, et al. Intrahepatic cholestasis of pregnancy: spontaneous vs in vitro fertilization. *Euroasian J Hepatogastroenterology* 2017; **7:** 126–129.
43 Carballu-Núñez E, González-Rodríguez L, González-Boubeta R, Alves-Pérez MT. Resultados perinatales en pacientes con colestasis gravídica. *Ginecologia y Obstetricia de Mexico* 2015; **83:** 776–784.
44 Çelik S, Çalışkan CS, Çelik H, Güçlü M, Başbuğ A. Predictors of adverse perinatal outcomes in intrahepatic cholestasis of pregnancy. *Ginekol Pol* 2019; **90:** 217–222.

45 Chen H, Zhou Y, Deng DR, Hao HY, Dang J, Li J. Intrahepatic cholestasis of pregnancy: Biochemical predictors of adverse perinatal outcomes. *J Huazhong Univ Sci Technol Med Sci* 2013; **33:** 412–417.

46 Demir BC, Güneş ES, Atalay MA. Intrahepatic cholestasis of pregnancy: Relationship between bile acid levels and maternal and fetal complications. *Turk J Obstet Gynecol* 2014; **11:** 148-152.

47 Erkenekli K, Iskender C, Onur Topçu H, Ensari T, Uygur D, Danisman N. Are Postprandial Bile Acid Levels Helpful in Predicting Perinatal Complications in Patients with Intrahepatic Cholestasis of Pregnancy? *Cukurova Med J* 2015; **40:** 212-220.

48 Feng C, Li W-J, He R-H, Sun X-W, Wang G, Wang L-Q. Impacts of different methods of conception on the perinatal outcome of intrahepatic cholestasis of pregnancy in twin pregnancies. *Sci Rep* 2018; **8:** 3985.

49 Frezza M**,** Centini G, Cammareri G, Le Grazie C, Di Padova C. S-adenosylmethionine for the treatment of intrahepatic cholestasis of pregnancy. Results of a controlled clinical trial. *Hepatogastroenterology* 1990; **37(**Suppl 2): 122–125.

50 Friberg AK, Zingmark V, Lyndrup J. Early induction of labor in high-risk intrahepatic cholestasis of pregnancy: what are the costs? *Arch Gynecol Obstet* 2016; **294:** 709–714.

51 Garcia-Flores J, Cañamares M, Cruceyra M, et al. Clinical value of maternal bile acid quantification in intrahepatic cholestasis of pregnancy as an adverse perinatal outcome predictor. *Gynecol Obstet Invest* 2015; **79:** 222–228.

52 Ge X, Xu YQ, Huang SH, et al. Intrahepatic cholestasis of pregnancy and fetal outcomes: a prospective birth cohort study. *Zhonghua Liu Xing Bing Xue Za Zhi* 2016; **37:** 187–191.

53 Glantz A, Marschall HU, Lammert F, Mattsson LA. Intrahepatic cholestasis of pregnancy: a randomized controlled trial comparing dexamethasone and ursodeoxycholic acid. *Hepatology* 2005; **42:** 1399–1405.

54 Heinonen S, Kirkinen P. Pregnancy outcome with intrahepatic cholestasis. *Obstet Gynecol* 1999; **94:** 189–193.

55 Herrera CA, Manuck TA, Stoddard GJ, et al. Perinatal outcomes associated with intrahepatic cholestasis of pregnancy. *J Matern Fetal Neonatal Med* 2018; **31:** 1913–1920.

56 Jain R, Suri V, Chopra S, Chawla YK, Kumara Kohli K. Obstetric cholestasis: outcome with active management. *J Obstet Gynaecol Res* 2013; **39:** 953–959.

57 Jin J, Pan S, Huang L, Yu Y, Zhong M, Zhang G. Risk factors for adverse fetal outcomes among women with early-versus late-onset intrahepatic cholestasis of pregnancy. *Int J Gynecol Obstet* 2015; **128:** 236–240.

58 Joutsiniemi T, Timonen S, Linden M, Suvitie P, Ekblad U. Intrahepatic cholestasis of pregnancy: observational study of the treatment with low-dose ursodeoxycholic acid. *BMC Gastroenterol* 2015; **15:** 92.

59 Kawakita T, Parikh LI, Ramsey PS, et al. Predictors of adverse neonatal outcomes in intrahepatic cholestasis of pregnancy. *Am J Obstet Gynecol* 2015; **213:** 570.e1–e8.

60 Koroglu N, Tayyar A, Nur Tola E et al. Increased levels of the novel heptokine fetuin B in patients with intrahepatic cholestasis of pregnancy. *J Matern Fetal Neonatal Med* 2019; **32:** 1620–1625.

61 Kowalska-Kańka A, Maciejewski T, Niemiec KT. The concentrations of bile acids and erythropoietin in pregnant women with intrahepatic cholestasis and the state of the fetus and newborn. *Med Wieku Rozwoj* 2013; **17:** 232–245.

62 Labbe C, Delesalle C, Creveuil C, Dreyfus M. Cholestases intrahepatiques gravidiques (CIG) précoces et tardives: étude des complications materno-fœtales. *Gynécologie Obs Fertil Sénologie* 2018; **46:** 388–394.

63 Li L, Cong L, Chen Y, Yang Y. Risk factors for adverse fetal outcomes in patients with intrahepatic cholestasis of pregnancy. *Biomedical Res* 2017; **28:** 9193–9197.

64 Lin J, Gu W, Hou Y. Diagnosis and prognosis of early-onset intrahepatic cholestasis of pregnancy: a prospective study. *J Matern Fetal Neonatal Med* 2019; **32:** 997–1003.

65 Liu Y, Qiao F, Liu H, Liu D. Ursodeoxycholic acid in the treatment of intrahepatic cholestasis of pregnancy. *J Huazhong Univ Sci Technolog Med Sci* 2006; **26:** 350–352.

66 Lu J, Kuang J, Cheng X. Study on the relationship between prenatal monitoring index in intrahepatic cholestasis pregnancy and perinatal prognosis. *Zhonghua Liu Xing Bing Xue Za Zhi* 2014; **35:** 1281–1283.

67 Mahey R, Agarwal N, Kriplani A, Saraya A, Garg P. Role of serum bile acids in diagnosis of intrahepatic cholestasis of pregnancy and effect of ursodeoxycholic acid therapy on bile acids and perinatal outcome. *Int J Gynecol Obstet* 2009; **107:** S444–S445.

68 Oztas E, Erkenekli K, Ozler S, et al. Can routine laboratory parameters predict adverse pregnancy outcomes in intrahepatic cholestasis of pregnancy? *J Perinat Med* 2015; **43:** 667–674.

69 Nicastri PL, Diaferia A, Tartagni M, Loizzi P, Fanelli M. A randomised placebo-controlled trial of ursodeoxycholic acid and S-adenosylmethionine in the treatment of intrahepatic cholestasis of pregnancy. *Br J Obstet Gynaecol* 2005; **105:** 1205–1207.

70 Pata O, Vardarelı E, Ozcan A, et al. Intrahepatic cholestasis of pregnancy: correlation of preterm delivery with bile acids. *Turkish J Gastroenterol* 2011; **22:** 602–605.

71 Proehl S, Piacquadio K, Getahun D, Fassett M, Ramos GS, Trivedi N. Change to bile acid level and risk of stillbirth inintrahepatic cholestasis of pregnancy. *Am J Obstet Gynecol* 2017; **216:** S447.

72 Raddatz LM, Jennewein L, Pulcer P, Louwen F. Pregnancy outcome in patients with intrahepatic cholestasis of pregnancy in a single German center. *Eur J Obstet Gynecol Reprod Biol* 2019; **234:** E59.

73 Reyihanguli M, Liu HY, Han WH et al. Influence of intrahepatic cholestasis during pregnancy on the incidence of preterm birth. *Zhonghua Liu Xing Bing Xue Za Zhi* 2017; **38:** 1415–1418.

74 Riikonen S, Savonius H, Gylling H, Nikkilä K, Tuomi AM, Miettinen TA. Oral guar gum, a gel-forming dietary fiber relieves pruritus in intrahepatic cholestasis of pregnancy. *Acta Obstet Gynecol Scand* 2000; **79:** 260–264.

75 Rioseco AJ, Ivankovic MB, Manzur A, et al. Intrahepatic cholestasis of pregnancy: a retrospective case-control study of perinatal outcome. *Am J Obstet Gynecol* 1994; **170**: 890–895.

76 Sargin Oruç A, Seçkin B, Özcan N, Özyer S, Uzunlar Ö, Danişman N. Role of postprandial bile acids in prediction of perinatal outcome in intrahepatic cholestasis of pregnancy. *J Obstet Gynaecol Res* 2014; **40:** 1883–1889.

77 Singla S, Aggarwal P, Mittal A. Study to show the effect of intrahepatic cholestasis of pregnancy on perinatal outcome in uncomplicated pregnancy. *J Evolution Med Dent Sci* 2016; **5:** 4964–4966.

78 Temel Yüksel I, Aslan Çetin B, Köroğlu N, Aydoğan Mathyk B, Erdem B. Inflammatory marker YKL-40 levels in intrahepatic cholestasis of pregnancy. *Gynecol Endocrinol* 2019; **35:** 635–637.

79 Türkmen GG, Timur H, Yilmaz Z, et al. Effect of intrahepatic cholestasis of pregnancy on maternal serum screening tests. *J Neonatal Perinatal Med* 2016; **9:** 411–415.

80 Vural Yilmaz Z, Gencosmanoglu Turkmen G, Daglar K, Yılmaz E, Kara O, Uygur D. Elevated red blood cell distribution width is associated with intrahepaticcholestasis of pregnancy. *Ginekol Pol* 2017; **88:** 75–80.

81 Yayla Abide Ç, Vural F, Kılıççı Ç, et al. Can we predict severity of intrahepatic cholestasis of pregnancy using inflammatory markers? *Turk J Obstet Gynecol* 2017; **14:** 160–165.

82 Zapata R**,** Sandoval L, Palma J, et al. Ursodeoxycholic acid in the treatment of intrahepatic cholestasis of pregnancy. A 12-year experience. *Liver Int* 2005; **25:** 548–554.

83 Zecca E, De Luca D, Marras M, Caruso A, Bernardini T, Romagnoli C. Intrahepatic cholestasis of pregnancy and neonatal respiratory distress syndrome. *Pediatrics* 2006; **117:** 1669–1672.

84 Zhang K, He J, Dong M. Relationship between umbilical artery Doppler waveform analysis and perinatal prognosis in women with intrahepatic cholestasis of pregnancy. *Int J Gynaecol Obstet* 2010; **111:** 187–188.

85 Zhang Y, Hu L, Cui Y, et al. Roles of PPARγ/NF-κB signaling pathway in the pathogenesis of intrahepatic cholestasis of pregnancy. *PLoS One* 2014; **9:** e87343.

86 Zhang L, Liu X-H, Qi H-B, et al. Ursodeoxycholic acid and S-adenosylmethionine in the treatment of intrahepatic cholestasis of pregnancy: a multi-centered randomized controlled trial. *Eur Rev Med Pharmacol Sci* 2015; **19:** 3770–3776.

87 Zhou F, Zhang L, He MM, Liu ZF, Gao BX, Wang XD. Corticotropin-releasing hormone expression in patients with intrahepatic cholestasis of pregnancy after ursodeoxycholic acid treatment: an initial experience. *Curr Med Res Opin* 2014; **30:** 1529–1535.

88 Ai Y, Liu SY. Comparison of three methods in the treatment of intrahepatic cholestasis of pregnancy. *J Practical Obstet Gynecol* 2002; **18:** 20–22.

89 Diaferia A, Nicastri PL, Tartagni M, Loizzi P, Iacovizzi C, Di Leo A. Ursodeoxycholic acid therapy in pregnancy women with cholestasis. *Int J Gynecol Obstet* 1996; **52:** 133–140.

90 Floreani A, Paternoster D, Melis A, Grella PV. S-adenosylmethionine versus ursodeoxycholic acid in the treatment of intrahepatic cholestasis of pregnancy: preliminary results of a controlled trial. *Eur J Obstet Gynecol Repro Biol* 1996; **67:** 109–113.

91 Joutsiniemi T, Timonen S, Leino R, Palo P, Ekblad U. Ursodeoxycholic acid in the treatment of intrahepatic cholestasis of pregnancy: a randomized controlled trial. *Arch Gynecol Obstet* 2014; **289:** 541–547.

92 Palma J, Reyes H, Ribalta J, et al. Ursodeoxycholic acid in the treatment of cholestasis of pregnancy: a randomized double-blind study controlled with placebo. *J Hepatol* 1997; **27:** 1022–1028.
